# Multivitamins in the prevention of cancer and cardiovascular disease: the COcoa Supplement and Multivitamin Outcomes Study (COSMOS) randomized clinical trial

**DOI:** 10.1093/ajcn/nqac056

**Published:** 2022-03-16

**Authors:** Howard D Sesso, Pamela M Rist, Aaron K Aragaki, Susanne Rautiainen, Lisa G Johnson, Georgina Friedenberg, Trisha Copeland, Allison Clar, Samia Mora, M Vinayaga Moorthy, Ara Sarkissian, Jean Wactawski-Wende, Lesley F Tinker, William R Carrick, Garnet L Anderson, JoAnn E Manson, COSMOS Research Group, JoAnn E Manson, Howard D Sesso, Pamela M Rist, Susanne Rautiainen Lagerstrom, Shari S Bassuk, Lu Wang, Aditi Hazra, Heike Gibson, Meryl S LeBoff, Samia Mora, Olivia I Okereke, Deirdre K Tobias, Nancy R Cook, Paulette D Chandler, William Christen, Georgina Friedenberg, Trisha Copeland, Jasmah Hanna, Allison Clar, Denise D'Agostino, Manickavasagar Vinayagamoorthy, Heike Gibson, Eunjung Kim, Martin Van Denburgh, Gregory Kotler, Chunying Li, Vadim Bubes, Ara Sarkissian, Doug Smith, Eduardo C Pereira, Melvyn Okeke, Elise Roche, David Bates, Claire Ridge, Alexandra Phillips, Brielle Salvo, Annalee Wilson, Leah Hall, Jimaldy Baez, Young-Hwan Sim, Hayara Cardoso, Gabriel Senor, Connor Rudnicki, Hanh Huynh, Viviane Nguyen, Nicholas Terrell, Beth A Holman, Joseph Walter, Lisa Fields Johnson, Amy Casarella, Julia O'Connell, William Christen, Susanne Rautiainen Lagerstrom, Luc Djoussé, Paulette D Chandler, Aditi Hazra, Deidre K Tobias, Zareen M Farukhi, Lu Wang, Xuehong Zhang, Kenneth Breen, George V Menjin Jr, Rolando Rodriguez, Shamikhah Curry, Samia Mora, Leah Arsenault, Olubunmi Solano, Alison Weinberg, Jennifer Coates, Matthew Kilroe, Lincoln Zernicke, Katelyn Hasson, Karen Matthew, Samia Mora, Chris Pfeffer, Julie Duszlak, David Bates, Vincent Guzman, Josue Falcon, Alex Romero, Henry Kupets, Frank Cortez, James C LeSuer, Andrea Hrbek, Eileen Bowes, Philomena Quinn, Megan Mele, Garnet L Anderson, Lisa Johnson, Leslie F Tinker, Aaron K Aragaki, Megan Herndon, Sue L Mann, Mary Pettinger, Rebecca P Hunt, Bill Carrick, Kate Szyperski, Lori Proulx-Burns, Elizabeth Burrows, Marian Limacher, Judith Hsia, Ganesh Asaithambi, Muhib Khan, Nandakumar Nagaraja, Lenore C Ocava, Jana Wold, Brian Silver, Stephanie Connelly, Gretchen Van Lom, Cris Garvida, Kathy Hightower, Patricia Spaulding, Wei Lin, Jenny Schoenberg, Patti Olee, Lawrence S Cohen, Theodore Colton, I Craig Henderson, Stephen Hulley, Alice H Lichtenstein, Eugene R Passamani, Rebecca A Silliman, Nanette Wenger, Shari E Ludlam, Hagen Schroeter, Michael Fare, Javier Ottawani, Catherine Kwik-Uribe, Cassandra Arnaiz, Ann Costanza, John Greene, Paul Hennessey, Sarma Vadlamani, Mallik Karmsetty, Paul Martini, Jan-Willem van Klinken, Alpa Shah, Lori Stern

**Affiliations:** Division of Preventive Medicine, Brigham and Women's Hospital and Harvard Medical School, Boston, MA, USA; Department of Epidemiology, Harvard T.H. Chan School of Public Health, Boston, MA, USA; Division of Preventive Medicine, Brigham and Women's Hospital and Harvard Medical School, Boston, MA, USA; Department of Epidemiology, Harvard T.H. Chan School of Public Health, Boston, MA, USA; Division of Public Health Sciences, Fred Hutchinson Cancer Research Center, Seattle, WA, USA; Division of Preventive Medicine, Brigham and Women's Hospital and Harvard Medical School, Boston, MA, USA; Department of Global Public Health, Karolinska Institutet, Stockholm, Sweden; Division of Public Health Sciences, Fred Hutchinson Cancer Research Center, Seattle, WA, USA; Division of Preventive Medicine, Brigham and Women's Hospital and Harvard Medical School, Boston, MA, USA; Division of Preventive Medicine, Brigham and Women's Hospital and Harvard Medical School, Boston, MA, USA; Division of Preventive Medicine, Brigham and Women's Hospital and Harvard Medical School, Boston, MA, USA; Division of Preventive Medicine, Brigham and Women's Hospital and Harvard Medical School, Boston, MA, USA; Division of Cardiovascular Medicine, Brigham and Women's Hospital and Harvard Medical School, Boston, MA, USA; Division of Preventive Medicine, Brigham and Women's Hospital and Harvard Medical School, Boston, MA, USA; Division of Preventive Medicine, Brigham and Women's Hospital and Harvard Medical School, Boston, MA, USA; Department of Epidemiology and Environmental Health, School of Public Health and Health Professions, State University of New York at Buffalo, Buffalo, NY, USA; Division of Public Health Sciences, Fred Hutchinson Cancer Research Center, Seattle, WA, USA; Division of Public Health Sciences, Fred Hutchinson Cancer Research Center, Seattle, WA, USA; Division of Public Health Sciences, Fred Hutchinson Cancer Research Center, Seattle, WA, USA; Division of Preventive Medicine, Brigham and Women's Hospital and Harvard Medical School, Boston, MA, USA; Department of Epidemiology, Harvard T.H. Chan School of Public Health, Boston, MA, USA

**Keywords:** multivitamin, cancer, randomized clinical trial, cardiovascular disease, cocoa extract, flavanols

## Abstract

**Background:**

Although older adults commonly take multivitamin-multimineral (MVM) supplements to promote health, evidence on the use of daily MVMs on invasive cancer is limited.

**Objectives:**

The study objective was to determine if a daily MVM decreases total invasive cancer among older adults.

**Methods:**

We performed a randomized, double-blind, placebo-controlled, 2-by-2 factorial trial of a daily MVM and cocoa extract for prevention of cancer and cardiovascular disease (CVD) among 21,442 US adults (12,666 women aged ≥65 y and 8776 men aged ≥60 y) free of major CVD and recently diagnosed cancer. The intervention phase was from June 2015 through December 2020. This article reports on the MVM intervention. Participants were randomly assigned to daily MVM or placebo. The primary outcome was total invasive cancer, excluding nonmelanoma skin cancer. Secondary outcomes included major site-specific cancers, total CVD, all-cause mortality, and total cancer risk among those with a baseline history of cancer.

**Results:**

During a median follow-up of 3.6 y, invasive cancer occurred in 518 participants in the MVM group and 535 participants in the placebo group (HR: 0.97; 95% CI: 0.86, 1.09; *P* = 0.57). We observed no significant effect of a daily MVM on breast cancer (HR: 1.06; 95% CI: 0.79, 1.42) or colorectal cancer (HR: 1.30; 95% CI: 0.80, 2.12). We observed a protective effect of a daily MVM on lung cancer (HR: 0.62; 95% CI: 0.42, 0.92). The composite CVD outcome occurred in 429 participants in the MVM group and 437 participants in the placebo group (HR: 0.98; 95% CI: 0.86, 1.12). MVM use did not significantly affect all-cause mortality (HR: 0.93; 95% CI: 0.81, 1.08). There were no safety concerns.

**Conclusions:**

A daily MVM supplement, compared with placebo, did not significantly reduce the incidence of total cancer among older men and women. Future studies are needed to determine the effects of MVMs on other aging-related outcomes among older adults. This trial is registered at www.clinicaltrials.gov as NCT02422745.

See corresponding article on page 1490.

## Introduction

Multivitamin-multimineral (MVM) formulations, which lack a universal definition (1) but typically provide ≥100% of the recommended daily value of most essential vitamins and minerals (2), are the most common dietary supplement taken in the United States and other high-income countries (3–5). Approximately one-third of US adults report regular MVM use (3, 4, 6), with an even greater prevalence among older adults (7), women (8), and cancer survivors (7, 9–11). Many US adults continue to take MVMs for general health and well-being (12, 13) or to reduce the risk of chronic diseases (14) despite inconsistency among observational studies examining long-term MVM use and risk of cancer (15–24) and cardiovascular disease (CVD) (18, 25–29).

Large-scale trials testing broad-based MVMs on clinical outcomes remain limited to the Physicians’ Health Study (PHS) II of 14,641 men aged ≥50 y, for which a typical MVM had a modest, significant 8% reduction in total cancer (30) and no significant reductions in colorectal, lung, and bladder cancer after 11 y of follow-up. In PHS II, MVM use significantly reduced total cancer by 27% among 1312 men with a baseline history of cancer. There was a potentially stronger effect of an MVM among older men aged ≥70 y, with a significant 18% reduction in cancer (30, 31).

The persistent use of MVMs, the broad public implications of even modest efficacy on cancer and other aging-related outcomes, and the paucity of data among women warranted a new clinical trial to improve our understanding of the health effects of MVM use in older adults. Therefore, we conducted the COcoa Supplement and Multivitamin Outcomes Study (COSMOS), a large-scale, randomized, double-blind, placebo-controlled trial testing the effects of a common MVM in the prevention of cancer and CVD among women and men.

## Methods

**FIGURE 1:**
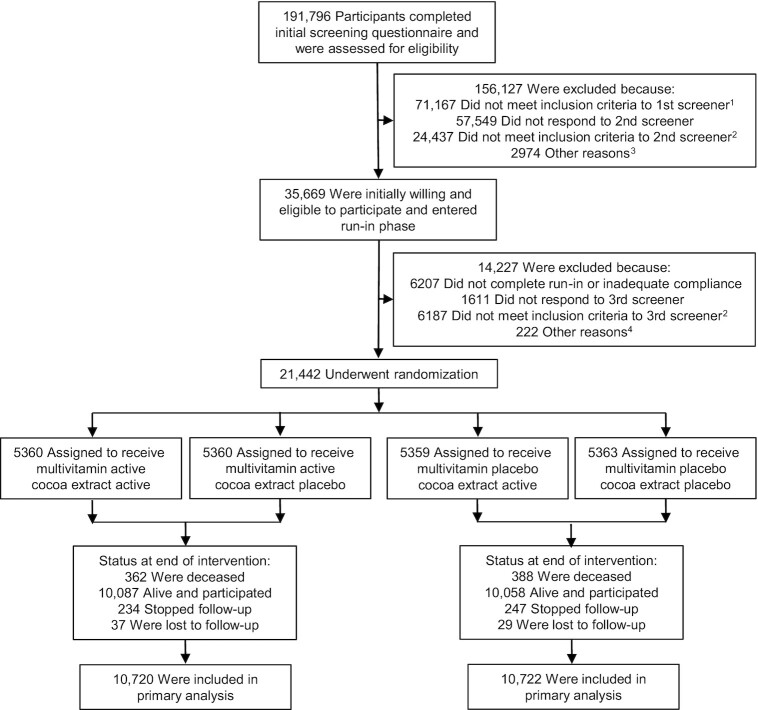
Screening, randomization, and follow-up of the participants. ^1^Eligibility was determined by medical history, age, and willingness to forego personal use of cocoa extract and multivitamin pills. ^2^Eligibility was determined by medical history, age, willingness to forego personal use of cocoa extract and multivitamin pills, caffeine sensitivity, and willingness to limit calcium and vitamin D supplement use. ^3^Included subjects who never completed the screening phase (*n* = 2914), eligibility could not be determined (*n* = 5), and enrollment goal already met (*n* = 55). ^4^Included subjects who never completed screening phase (*n* = 168) and enrollment goal already met (*n* = 54).

The COSMOS (clinicaltrials.gov #NCT02422745) is a randomized, double-blind, placebo-controlled, 2 × 2 factorial trial testing an MVM supplement (Centrum Silver®, supplied by Pfizer Consumer Healthcare, now a part of GSK Consumer Healthcare) and cocoa extract supplement [2 capsules/d containing 500 mg cocoa flavanols/d, including 80 mg (–)-epicatechin; supplied by Mars Edge] to prevent cancer and CVD in 21,442 US adults, including 12,666 women aged ≥65 y and 8776 men aged ≥60 y who were free of myocardial infarction (MI) and stroke and recently diagnosed cancer (except for nonmelanoma skin cancer) within the past 2 y (32). **Figure 1** summarizes the overall COSMOS trial design.

Briefly, an embedded recruitment approach was conducted from June 2015 to March 2018 with mailings to active participants in the Women's Health Initiative (WHI) Extension Study (33), mailings by Brigham and Women's Hospital (BWH) to women and men contacted for but not randomly assigned to the VITamin D and OmegA-3 TriaL (VITAL) (34), mass mailings, and mailings to volunteers who heard about the study through various sources (**Supplementary Methods**). To be eligible, participants agreed to forego personal MVM and cocoa extract supplement use during the trial, limit vitamin D to ≤1000 IU/d and calcium to ≤1200 mg/d from all supplemental sources, and complete at least a 2-mo placebo run-in phase taking ≥75% of the study pills. Of 191,796 participants completing a brief initial screening questionnaire, 35,669 eligible, willing, and consenting participants began a placebo run-in (35).

From April 2016 to March 2018, 21,442 participants meeting all eligibility requirements were randomly assigned in equal proportions to *1*) active MVM and active cocoa extract, *2*) active MVM and cocoa extract placebo, *3*) active cocoa extract and MVM placebo, or *4*) both placebos using a computer-generated permuted block approach blinded to trial investigators and stratified by sex (women, men), age (separate 5-y age groups for women and men), and recruitment source (WHI, BWH) in blocks of 12. Prior to randomization, 6867 (32.0%) of randomized participants provided biospecimens, of whom 2050 additionally provided at least 1 follow-up biospecimen at 1, 2, and/or 3 y follow-up. Mars Edge supported the measurement of 25-hydroxyvitamin D [25(OH)D], vitamin B-12, and folate concentrations in a random subset of 399 participants to evaluate compliance with the MVM intervention during follow-up.

Participants received follow-up questionnaires, with calendar packs containing study pills, at 6 mo and 12 mo following randomization and semi-annually thereafter to assess compliance with randomized treatments, use of nontrial MVMs and/or cocoa supplements, potential side effects of the interventions, updated medical history, and other relevant lifestyle, clinical, and dietary risk factors. The randomized treatments continued through 31 December 2020, ending as scheduled, with a median (IQR) treatment period of 3.6 (3.2–4.2) y.

All participants provided written informed consent before enrollment in the trial, and trial activities were overseen by the Human Subjects Committee at BWH/Mass General Brigham.

### Primary and secondary outcomes

For the MVM intervention, the primary outcome was total invasive cancer, excluding nonmelanoma skin cancer. There were 14 secondary outcomes and 4 other outcomes that mostly consisted of subtypes of CVD or cancer. Secondary outcomes included key site-specific cancers (including breast, colorectal, and lung cancer), all-cause death, a composite total CVD outcome (including incident MI, stroke, coronary revascularization, cardiovascular mortality, carotid artery surgery, peripheral artery surgery, and unstable angina requiring hospitalization), a combined outcome of CVD and all-cause mortality, and individual cardiovascular events comprising the composite total CVD outcome.

Participants reporting a study outcome signed a medical record release form to request related medical records (36). Outcomes were adjudicated by medical record review by a committee of physicians and trained investigators and staff blinded to treatment assignment. Incident cancers were confirmed by a pathology report that substantiated a malignant primary invasive cancer at any location other than nonmelanoma skin cancer (36); all histologic types and anatomic subsites were included. Noncancerous colorectal polyps, atypical benign breast disease, in situ cancers, and other premalignant benign conditions were excluded. MI, stroke (37, 38), coronary revascularization (36), and other vascular outcomes were confirmed using established clinical criteria (see Supplementary Methods for details). For participants determined to be deceased, we contacted next of kin to request permission to obtain medical records and a copy of the death certificate. For WHI participants, death certificates were alternatively requested from the state where the participant died. An outcomes committee reviewed records to assign cause of death. If records were unavailable (or participants were lost to follow-up), the National Death Index Plus was searched for cause of death according to the death certificate information. Analyses included only confirmed outcomes.

Throughout trial follow-up, we collected information on potential side effects and other self-reported nonmonitored outcomes such as skin cancer, colon or rectal polyps, other cardiovascular outcomes, and endocrine, metabolic, and digestive system diseases.

### Statistical analyses

Our primary analyses were based on the intention-to-treat principle for time-to-first-event data (39). Cox proportional hazards models estimated HRs using an indicator variable for MVM assignment and stratifying the baseline hazard functions by sex, age group, recruitment source, and cocoa extract intervention arm. The trial was designed for 22,000 participants with ≥90% power to detect a 14% relative hazard reduction in total cancer. Models were constructed for each clinical outcome, where person-time for each outcome was counted as days from randomization to the first post-randomization diagnosis of the designated outcome. Follow-up was censored at date of last contact, death, or end of the trial on 31 December 2020, whichever came first.

Kaplan-Meier cumulative incidence curves, cumulative HRs, interactions between randomization groups with trial time, and analyses that excluded the first 2 y (for cancer endpoints) or 1 y (for CVD endpoints) of follow-up assessed whether treatment effects varied over time (40). Ten subgroup analyses examined effect modification by a priori (concurrent cocoa extract randomization assignment, sex, age, history of cancer, use of statins or aspirin) and post hoc (smoking status, fruit and vegetable intake at baseline, use of dietary supplements or multivitamins prior to randomization) factors. Statistical significance (*P* ≤ 0.05) was assessed with 2-sided *P* values. We did not adjust *P* values or CIs for multiple testing. Secondary outcomes mostly constituted subtypes of cancer or CVD, and numbered fewer than 20. Results for secondary and exploratory outcomes and those for subgroup analyses should therefore be interpreted with caution and considered hypothesis generating. At the nominal 0.05 level, we would expect less than 1 interaction and 1 secondary outcome to be significant by chance alone.

The **Supplementary Results** provides details on per-protocol analyses and analyses of the blood nutrient biomarkers among a subset of individuals with baseline and follow-up blood samples. Finally, we compared self-reports of nonmonitored outcomes and potential side effects by intervention group. Analyses were performed using SAS version 9.4 (SAS Institute).

## Results

A total of 21,442 individuals were randomly assigned into the COSMOS trial (Figure 1). The baseline characteristics of the study participants were similar between the MVM and placebo groups (**Table 1** and **Supplementary Table 1**). The mean (SD) age of the participants was 72.1 (6.6) y and 59.1% were women. T total of 3550 (16.6%) participants had a baseline history of cancer. Prior to randomization, 41.2% reported use of an MVM, 21.4% reported consuming >1000 IU vitamin D/d from supplements, 5.0% reported consuming >1200 mg calcium/d from supplements, and 35.0% reported consuming <4 servings of fruit and vegetables per day. A large proportion were former smokers (41.3%), although only 4.0% were current smokers.

**TABLE 1:** Characteristics of the participants at baseline, according to randomized assignment^1^

	Total (*n* = 21,442)		Multivitamin (*n* = 10,720)		Placebo (*n* = 10,722)	
	*n*	(%)	*n*	(%)	*n*	(%)
Female sex	12,666	(59.1)	6338	(59.1)	6328	(59.0)
Age, mean ± SD, y	72.1± 6.6		72.1± 6.6		72.1± 6.6	
Hispanic/Latino^2^	544	(2.6)	284	(2.8)	260	(2.5)
Race^2^						
White	19,294	(90.0)	9628	(89.8)	9666	(90.2)
African American	1131	(5.3)	568	(5.3)	563	(5.3)
Asian/Pacific Islander	499	(2.3)	258	(2.4)	241	(2.2)
American Indian/Alaska Native	59	(0.3)	37	(0.3)	22	(0.2)
Multiracial/other/unknown or not reported	459	(2.1)	229	(2.1)	230	(2.1)
Education						
High school diploma/GED or less	2296	(10.8)	1180	(11.1)	1116	(10.5)
Attended or graduated from college	8685	(40.9)	4315	(40.7)	4370	(41.1)
Post-college	10,241	(48.3)	5104	(48.2)	5137	(48.4)
Smoking status						
Never	11,565	(54.7)	5808	(54.9)	5757	(54.5)
Past	8731	(41.3)	4345	(41.1)	4386	(41.5)
Current	835	(4.0)	417	(3.9)	418	(4.0)
Multivitamin use before run-in	8795	(41.2)	4413	(41.3)	4382	(41.0)
Vitamin D from supplements before run-in						
None	7960	(37.6)	4012	(37.9)	3948	(37.3)
≤1000 IU/d	8670	(41.0)	4331	(40.9)	4339	(41.0)
>1000 IU/d	4536	(21.4)	2238	(21.2)	2298	(21.7)
Calcium from supplements before run-in						
None	10,917	(51.5)	5483	(51.8)	5434	(51.3)
≤1200 mg/d	9200	(43.4)	4596	(43.4)	4604	(43.5)
>1200 mg/d	1066	(5.0)	516	(4.9)	550	(5.2)
History of diabetes	2864	(13.4)	1415	(13.2)	1449	(13.5)
History of high blood pressure	12,423	(58.1)	6153	(57.6)	6270	(58.6)
Statin use	8911	(42.1)	4464	(42.2)	4447	(41.9)
Aspirin use	10,379	(48.9)	5168	(48.7)	5211	(49.1)
History of revascularization (CABG/PCI)	862	(4.0)	426	(4.0)	436	(4.1)
History of unstable angina	374	(1.8)	194	(1.8)	180	(1.7)
History of carotid artery surgery/stenting	93	(0.4)	44	(0.4)	49	(0.5)
History of peripheral artery surgery/stenting	144	(0.7)	81	(0.8)	63	(0.6)
History of heart failure	364	(1.7)	188	(1.8)	176	(1.7)
History of cancer excluding nonmelanoma skin cancer	3550	(16.6)	1813	(16.9)	1737	(16.2)

1 *n* = 21,442. Percentages may not sum to 100 because of rounding. Data on age and sex were complete. Data on other characteristics were available for ≥98.5% of the trial participants. Multivitamin refers to multivitamin-multimineral which was tested in COSMOS. CABG/PCI, coronary artery bypass graft and percutaneous coronary intervention; COSMOS, COcoa Supplement and Multivitamin Outcomes Study; GED, General Educational Development.

2 Ethnic group and race were self-reported by participants. Multiracial participants self-identified with >1 race. Participants of other race or unknown race self-identified with those categories.

**FIGURE 2:**
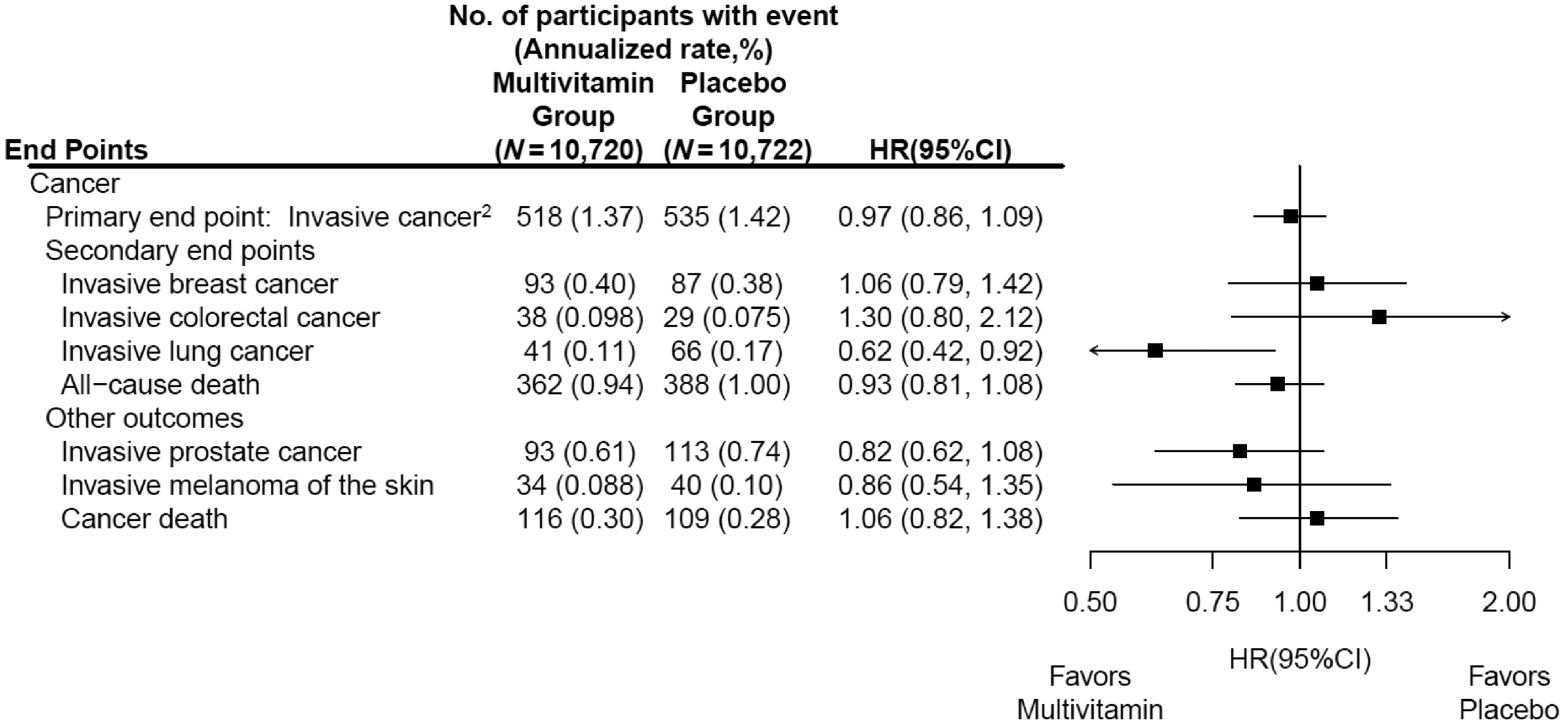
HRs and 95% CIs^1^ for the primary and secondary cancer outcomes, according to randomized assignment, in intention-to-treat analyses. ^1^Summary statistics were from Cox regression models that stratified baseline hazard functions by cocoa extract trial randomization group, age, sex, and recruitment cohort. Analyses were not adjusted for multiple comparisons. ^2^This outcome was a composite of invasive cancers of any site other than nonmelanoma skin cancer.

**FIGURE 3:**
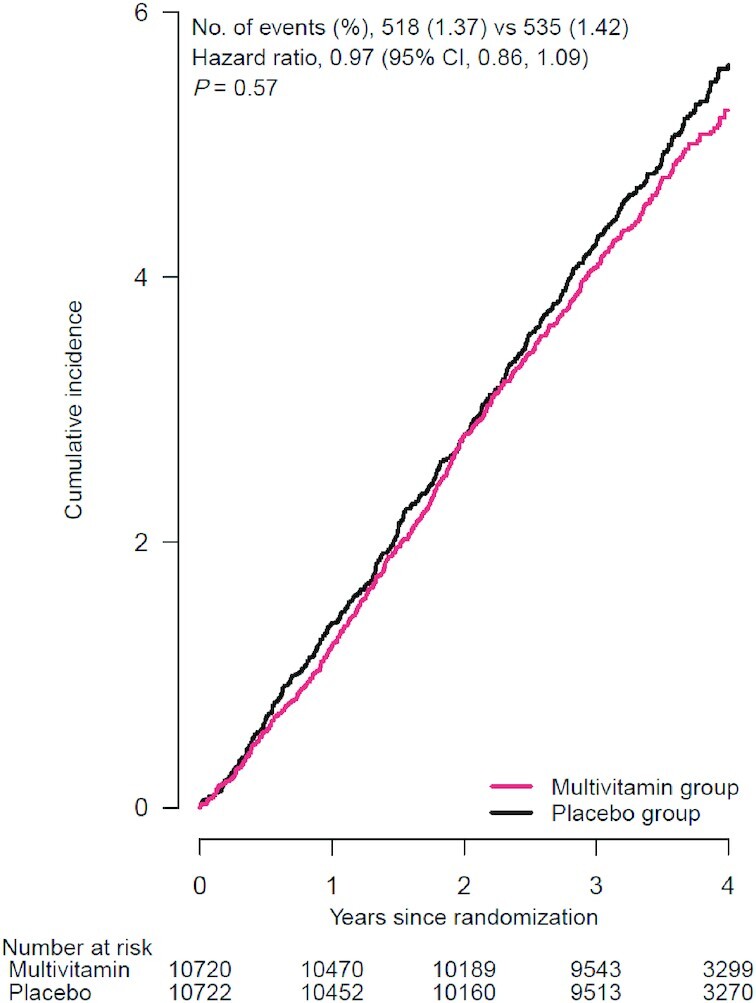
Cumulative incidence rates of invasive cancer events,^1^ according to year of follow-up, in multivitamin group and placebo group. ^1^Primary outcome: a composite of invasive cancers of any site other than nonmelanoma skin cancer. Summary statistics were from Cox regression models that stratified baseline hazard functions by cocoa extract trial randomization group, age, sex, and recruitment cohort (intention-to-treat analyses). *P* value was for the effect of randomization group, based on a stratified score (log-rank) test.

The mean follow-up time was 3.6 y (IQR: 3.2–4.2 y), with a total of 77,331 person-years of follow-up. The primary outcome of invasive cancer was confirmed in 1053 participants, including 180 cases of breast cancer (among women only), 67 cases of colorectal cancer, 107 cases of lung cancer, 206 cases of prostate cancer (among men only), and 74 cases of melanoma during the trial period. The annualized rates of total invasive cancer were 1.37% and 1.42% in the MVM and placebo groups, respectively. We observed no significant effect on the primary outcome of total invasive cancer (HR: 0.97; 95% CI: 0.86, 1.09) (**Figure 2**). The cumulative incidence curves for the MVM group and placebo groups were similar (log-rank *P* = 0.57) (**Figure 3**), as were the cumulative HRs (**Supplementary Figure 1**). When we excluded the first 2 y of follow-up to account for cancer latency, we observed no significant effect on the risk of invasive cancer for the MVM group compared with the placebo group (HR: 0.93; 95% CI: 0.77, 1.11).

**FIGURE 4:**
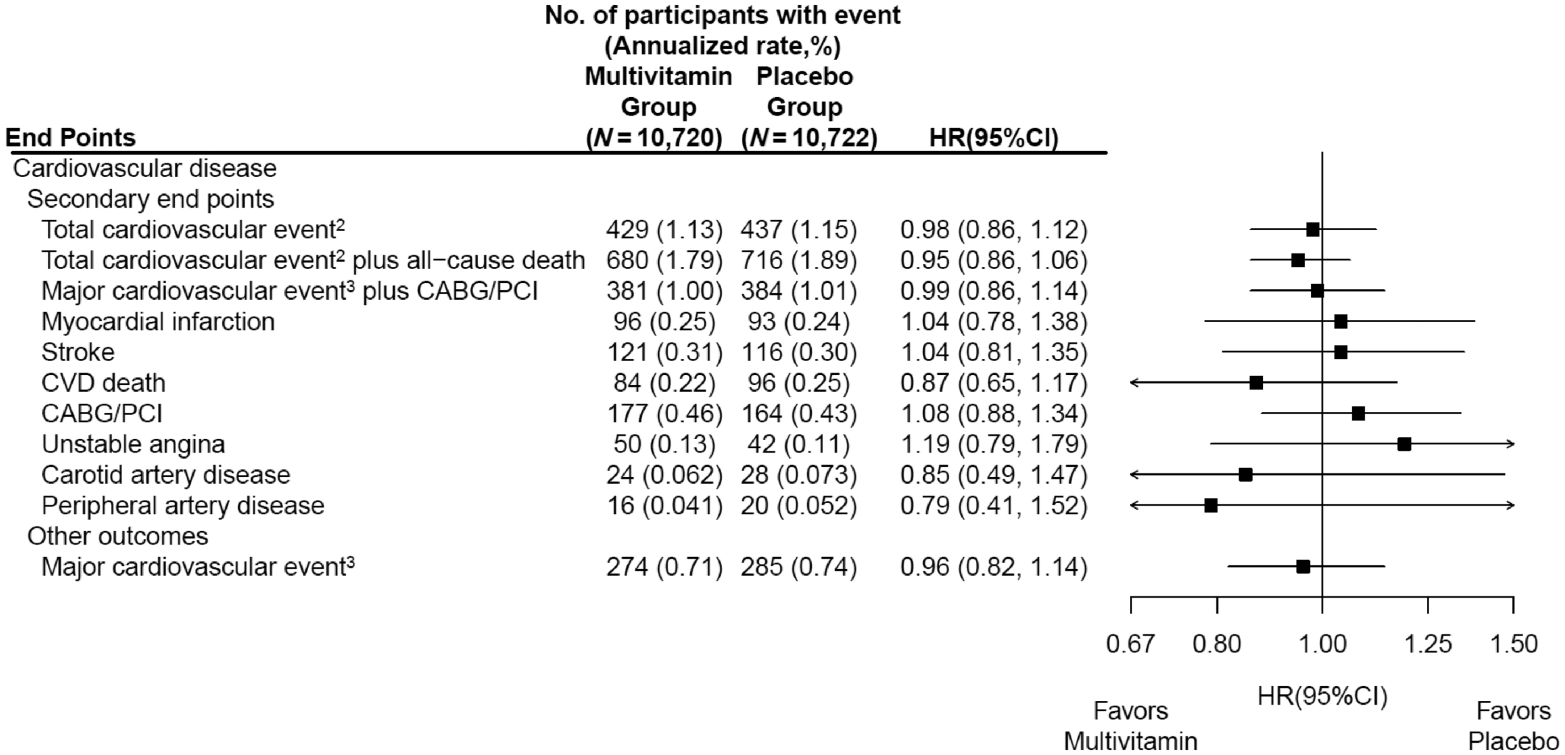
HRs and 95% CIs^1^ for secondary cardiovascular disease outcomes, according to randomized assignment, in intention-to-treat analyses. ^1^Summary statistics were from Cox regression models that stratified baseline hazard functions by cocoa extract trial randomization group, age, sex, and recruitment cohort. Analyses were not adjusted for multiple comparisons. ^2^This outcome was a composite of myocardial infarction, stroke, cardiovascular death, CABG/PCI, unstable angina including hospitalization, carotid artery surgery, and peripheral artery surgery or angioplasty. ^3^This outcome was a composite of myocardial infarction, stroke, and cardiovascular death. CABG/PCI, coronary artery bypass graft and percutaneous coronary intervention; CVD, cardiovascular disease.

We found no significant effect of a daily MVM on breast cancer (HR: 1.06; 95% CI: 0.79, 1.42), colorectal cancer (HR: 1.30; 95% CI: 0.80, 2.12), prostate cancer (HR: 0.82; 95% CI: 0.62, 1.08), or melanoma (HR: 0.86; 95% CI: 0.54, 1.35) (Figure 2). However, a daily MVM significantly reduced lung cancer compared with placebo (HR: 0.62; 95% CI: 0.42, 0.92) (Figure 2). We observed 225 cancer deaths during the trial, including 116 in the MVM group and 109 in the placebo group (HR: 1.06; 95% CI: 0.82, 1.38) (Figure 2). We did not observe a significant association between MVM use and all-cause mortality (HR: 0.93; 95% CI: 0.81, 1.08) or CVD mortality (HR: 0.87; 95% CI: 0.65, 1.17) (Figure 2 and **Figure 4**).

Results from the subgroup analyses for total invasive cancer are presented in **Supplementary Figure 2**. We did not find significant effect modification by any of the variables examined. Among 3550 participants with a baseline history of cancer, MVM use had no significant effect on total invasive cancer compared with placebo (HR: 0.97; 95% CI: 0.77, 1.23), and the effect was similar among those without a baseline history of cancer (HR: 0.97; 95% CI: 0.84, 1.11). There was a trend towards a lower risk of cancer among those who ever smoked (HR: 0.86; 95% CI: 0.72, 1.02), but this difference was not significantly different from that among never-smokers (HR: 1.08; 95% CI: 0.91, 1.28) (*P*-interaction = 0.06).

To facilitate comparison with the findings from PHS II, we performed sex-stratified analyses for total invasive cancer. MVM use was not associated with a significant reduction in the risk of total invasive cancer among men (HR: 0.94; 95% CI: 0.78, 1.13) or women (HR: 0.98; 95% CI: 0.84, 1.15) (*P*-interaction = 0.74) (Supplementary Figure 2).

Among participants who completed questionnaires assessing compliance, the percentage of participants who reported abstaining from use of nonstudy multivitamin supplements, abstaining from use of nonstudy vitamin D supplements >1000 IU/d, or having missed ≤8 d of MVM pills per month was >80% at 12, 24, and 36 mo and study closeout (**Supplementary Figure 3**). When we additionally considered those who did not return questionnaires to be noncompliant, total compliance among those taking active MVM compared with placebo was 71.0% and 68.6% at 36 mo (90.8% of randomized participants). Using our most restrictive definition of noncompliance, which incorporated compliance to both study-related pills, vitamin D intake, and follow-up questionnaires, analyses censoring at first report of noncompliance for total (HR: 0.97; 95% CI: 0.84, 1.12) and site-specific cancers paralleled the findings of our primary analyses (**Supplementary Figure 4**).

Among 399 participants with baseline and follow-up measures of serum 25(OH)D, vitamin B-12, and folate, concentrations of these biomarkers were balanced between the arms at baseline (Supplementary Table 1). Compared with the placebo group, the multivitamin group had significantly greater increases in serum 25(OH)D, vitamin B-12, and folate (**Supplementary Figures 5–7**; Supplementary Results).

Our secondary outcome of total CVD occurred in 866 individuals, including 189 first events of MI, 237 cases of stroke, 180 cases of cardiovascular death, 341 cases of coronary revascularization, 92 cases of unstable angina requiring hospitalization, 52 cases of carotid artery disease, and 36 cases of peripheral artery disease, with some participants experiencing multiple events. The annual event rates were 1.13% and 1.15% in the MVM and placebo groups, respectively. A daily MVM had no significant effect on total CVD (HR: 0.98; 95% CI: 0.86, 1.12) compared with placebo, which also extended to individual cardiovascular outcomes (Figure 4) and the combined outcome of CVD and all-cause mortality (HR: 0.95; 95% CI: 0.86, 1.06). Latency analyses excluding the first year of follow-up did not materially alter the findings for total CVD (HR: 1.02), all-cause mortality (HR: 0.92), or total CVD and all-cause mortality (HR: 0.95).

Those assigned to the active MVM had lower rates of self-reported stomach upset or pain (HR: 0.94; 95% CI: 0.90, 0.98), diarrhea (HR: 0.93; 95% CI: 0.90, 0.96), skin rash (HR: 0.95; 95% CI: 0.90, 0.99), and easy bruising (HR: 0.94; 95% CI: 0.90, 0.98) and a higher rate of gastrointestinal bleeding (HR: 1.17; 95% CI: 1.03, 1.32) compared with the placebo group. We found no other significant differences between the MVM and placebo groups for all other potential side effects or self-reported nonmonitored study outcomes (**Supplementary Figures 8** and **9**).

## Discussion

In this large-scale, randomized, placebo-controlled trial in older women and men with high compliance and follow-up, a daily MVM did not reduce the primary outcome of total invasive cancer. The lack of a significant effect of an MVM extended to those with a baseline history of cancer, in contrast to previous findings in men from PHS II (30). In addition, MVM supplementation had no significant effect on the secondary outcomes of total cardiovascular events, CVD death, or all-cause mortality.

A 2014 systematic review (41) and recommendation by the US Preventive Services Task Force (USPSTF) concluded that “the current evidence is insufficient to assess the balance of benefits and harms of multivitamins for the prevention of cardiovascular disease and cancer” (42). The USPSTF recently updated their systematic review (43) for new recommendations prior to the completion of COSMOS and these forthcoming recommendations should integrate the COSMOS trial results. Meanwhile, 70% of US adults aged ≥60 y take at least 1 dietary supplement daily (8), with MVMs remaining the most frequently used dietary supplement (3, 4), although use differs by socioeconomic and health-related factors (44) and MVMs are more likely to be used by those with a healthy lifestyle and diet (45). Therefore, there are major public health implications regarding MVM use in terms of perceived benefits and financial burden, with billions of dollars in annual global dietary supplement sales (46).

Individual vitamins and minerals may reduce cancer risk by protecting against free-radical damage and oxidation (47, 48), enhancing immune response (49, 50), blocking the formation of carcinogenic nitrosamines (51), and modulating tumor growth (48, 52, 53). However, an MVM cannot necessarily replicate the natural interactions of vitamins, minerals, and bioactive components in foods reflecting healthy dietary patterns, which are inversely associated with cancer (54, 55) and CVD (56, 57). Observational studies of MVM use and risk of cancer and CVD have been inconclusive (42, 58), likely reflecting uncontrolled confounding and selection bias. Further, it is important to consider the effect of an MVM on both total and individual cancer sites given the heterogeneity of cancer etiology and risk factors. In the Cancer Prevention Study II in more than 1 million US adults, MVM use was not associated with cancer mortality (59) but was inversely associated with incident colon cancer (60) and total mortality (20). Increasing duration of MVM use was inversely associated with colon cancer in the Nurses’ Health Study (21). However, there was little or no association between personal MVM use and risk of breast, colon, or other cancers in more than 160,000 WHI women followed for 8 y (18).

We tested a commonly used MVM with previously demonstrated safety in PHS II (30, 31), which included essential vitamins and minerals plus newly added lutein and lycopene (**Supplementary Table 2**). PHS II is the only other completed trial of a broad-based MVM among male physicians aged ≥50 y, with a longer median follow-up of 11 y, finding a modest, significant 8% reduction in total cancer (30). In COSMOS, we observed no significant association between MVM use and invasive cancer among men. Two-year latency analyses showed similar findings to the overall results, although power for these analyses was limited. Further, we found significant 38% reductions in lung cancer in COSMOS. However, PHS II did not observe a statistically significant association between MVM use and lung cancer. Among 3550 women and men in COSMOS with a baseline history of cancer, we did not replicate the significant 27% reduction on total cancer in PHS II. Despite a stronger 18% reduction in cancer among men aged ≥70 y in PHS II (31), COSMOS found no significant effect modification between MVM use and age on total cancer. Finally, in COSMOS, we found a similar lack of effect for an MVM on total CVD as in PHS II. Although both PHS II and COSMOS tested a common MVM, changes were made to the formulation between the 2 trials (Supplementary Table 2). We cannot directly determine whether differences in MVM formulations, along with other study design modifications, explain any differences in COSMOS compared with PHS II results.

Earlier supplement trials tested limited combinations of vitamins and minerals in contrast to COSMOS and PHS II. The Linxian trial tested a combination of β-carotene, vitamin E, and selenium for 6 y in 29,584 adults in rural China and found significant reductions of 9%, 13%, and 21% in mortality, cancer mortality, and gastric cancer mortality, respectively (61), which remained after 10 y of post-trial follow-up (62). The Heart Protection Study in 20,536 adults in the United Kingdom tested higher doses of the same nutrients but found no reductions in total or site-specific cancer or CVD during a mean follow-up of 5 y (63). Finally, the SU.VI.MAX trial randomly assigned 13,017 subjects to vitamin C, vitamin E, β-carotene, selenium, and zinc, with no overall effect on total cancer incidence or cancer mortality during a median follow-up of 7.5 y; however, they did observe a 31% decrease in total cancer incidence and a 37% decrease in cancer mortality in men only (64). Our placebo-controlled trial of a broad-based MVM cannot be extrapolated to the effects of individual nutrients or other MVM formulations.

Baseline nutritional status is another important consideration, as COSMOS participants may already have adequate or optimum nutrient intake for which MVM supplementation may offer limited benefits (65), whereas nutritionally vulnerable populations may improve nutritional status with broad-based MVMs (58, 66). We plan to expand evaluations of randomized MVM use, dietary status, changes in nutritional biomarkers, and cancer and CVD risk.

The COSMOS trial has several key strengths, including the enrollment of a large, older population of US adults with high rates of follow-up and compliance and medical record–adjudicated primary and secondary cancer and CVD outcomes. Utilizing a hybrid trial design (67), several ancillary studies are examining the effect of an MVM on cognition, eye health, changes in aging-related biomarkers, falls, and other clinically relevant outcomes to help us understand the overall balance of risks and benefits for MVM supplementation. Finally, we tested a commonly used MVM supplement to ensure broader generalizability of our findings.

Several limitations should be considered when interpreting our findings. First, the COSMOS intervention was relatively short to detect a potential small-to-moderate effect on cancer outcomes given the long duration of time typically required for nutritional interventions to potentially reduce cancer risk. Due to the short duration of COSMOS, we were unable to explore the impact of longer latency periods on our findings. Post-intervention follow-up for COSMOS may help clarify any longer-term effects on cancer, all-cause mortality, and other outcomes. Second, our secondary and exploratory analyses should be interpreted with caution, especially given an overall lack of effect of an MVM on the primary outcome of total invasive cancer. Third, we successfully leveraged existing cohorts with mass mailings to expedite recruitment and randomization of 21,442 participants into COSMOS. However, generalizability may be limited, with modest diversity of 10% non-Whites and 2.6% Hispanics plus healthy volunteer bias for participants willing and eligible to enroll in a mail-based clinical trial.

In conclusion, MVM supplementation did not reduce our primary outcome of total invasive cancer after 3.6 y of treatment in older women and men. MVM use also had no effect on cancer among those with a baseline history of cancer or on the secondary outcomes of total CVD. These findings do not support the regular use of MVMs for cancer or CVD prevention among generally healthy older men and women. Future studies should clarify the role of long-term MVM use on nutritional status and the balance of risks and benefits on cancer, CVD, and other aging-related outcomes.

## Supplementary Material

nqac056_Supplemental_File

## Data Availability

Data set(s) will be de-identified prior to release for sharing. We will make the data and associated documentation available to users only under a data-sharing agreement. Details on the availability of the study data to other investigators will be on our study website at https://cosmostrial.org/.

## Notes

The COcoa Supplement and Multivitamin Outcomes Study (COSMOS) is supported by an investigator-initiated grant from Mars Edge, a segment of Mars dedicated to nutrition research and products, which included infrastructure support and the donation of study pills and packaging. Pfizer Consumer Healthcare (now part of GSK Consumer Healthcare) provided support through the partial provision of study pills and packaging. COSMOS is also supported, in part, by grants AG050657, AG071611, EY025623, and HL157665 from the National Institutes of Health, Bethesda, MD. The Women's Health Initiative (WHI) is funded by the National Heart, Lung, and Blood Institute, NIH, US Department of Health and Human Services through contracts 75N92021D00001, 75N92021D00002, 75N92021D00003, 75N92021D00004, and 75N92021D00005. Neither Mars Edge nor Pfizer (GSK) Consumer Healthcare provided input regarding the design of the study, data analyses, interpretation of results, or manuscript development nor did they review, comment, or approve the manuscript.

Supplementary Methods, Supplementary Results, Supplementary Figures 1–9, and Supplementary Tables 1 and 2 are available from the “Supplementary data” link in the online posting of the article and from the same link in the online table of contents at https://academic.oup.com/ajcn/.

COSMOS was approved and overseen by the Institutional Review Board of Brigham and Women's Hospital/Mass General Brigham. The COSMOS website is www.cosmostrial.org.

HDS and PMR contributed equally as co-first authors.

Abbreviations used: BWH, Brigham and Women's Hospital; CABG, coronary artery bypass graft; COSMOS, COcoa Supplement and Multivitamin Outcomes Study; CVD, cardiovascular disease; MI, myocardial infarction; MVM, multivitamin-multimineral; PCI, percutaneous coronary intervention; PHS, Physicians’ Health Study; USPSTF, US Preventive Services Task Force; WHI, Women's Health Initiative; 25(OH)D, 25-hydroxyvitamin D.
