# Supplementary material for: Multivitamins in the prevention of cancer and cardiovascular disease: the COcoa Supplement and Multivitamin Outcomes Study (COSMOS) randomized clinical trial
Source: Am J Clin Nutr. 2022 Mar 16;115(6):1501–10. doi: 10.1093/ajcn/nqac056 (PMC9170475; doi:10.1093/ajcn/nqac056)
Supplement: nqac056_Supplemental_File [file nqac056_supplemental_file.docx]

**Online Supplementary Material**

**Multivitamins in the Prevention of Cancer and Cardiovascular Disease:**

**The COSMOS Randomized Clinical Trial**

Howard D. Sesso, ScD, MPH*,^1,2^ Pamela M. Rist, ScD*,^1,2^ Aaron K. Aragaki, MS,^3^ Susanne Rautiainen, PhD,^1,4^ Lisa G. Johnson, PhD,^3^ Georgina Friedenberg, MPH,^1^ Trisha Copeland, MS, RD,^1^ Allison Clar, BS,^1^ Samia Mora, MD, MHS,^1,5^ M. Vinayaga Moorthy, PhD,^1^ Ara Sarkissian, MA,^1^ Jean Wactawski-Wende, PhD,^6^ Lesley F. Tinker, PhD,^3^ William R. Carrick, BS,^3^ Garnet L. Anderson, PhD,^3^ and JoAnn E. Manson, MD, DrPH^1,2^ for the COSMOS Research Group

**Supplementary Methods.** Study Design, Primary and Secondary Outcomes, and Statistical Analyses – Per-protocol and Biomarker Data

**Supplementary Results.** Biomarker analyses

**Supplementary Table 1.** Characteristics of the participants at baseline, according to randomized assignment (n = 21,442)

**Supplementary Table 2.** Vitamins and minerals contained in the COcoa Supplement and Multivitamin Outcomes Trial (COSMOS) versus Physicians’ Health Study II (PHS II) multi-vitamin/multi-mineral interventions.

**Supplementary Figure 1.** Cumulative hazard ratios of invasive cancer events, according to year of follow-up, in multivitamin group and placebo group.

**Supplementary Figure 2.** Hazard ratios of the primary outcome according to subgroup, comparing multivitamin group with placebo group.

**Supplementary Figure 3.** Proportion of participants who reported abstaining from use of non-study multivitamins, abstaining from use of non-study vitamin D > 1000 IU/day, compliance to study pills, total compliance (adherence to all previous components), and total compliance and completion of follow-up questionnaires during the intervention phase (N=21,442).

**Supplementary Figure 4.** Hazard ratios and 95% confidence intervals for the primary and secondary cancer outcomes, according to randomized assignment, where follow-up of noncompliant participants was censored.

**Supplementary Figure 5.** Influence of multivitamin vs placebo on serum 25−hydroxyvitamin D (N = 399).

**Supplementary Figure 6.** Influence of multivitamin vs placebo on serum vitamin B_12_ (N = 399).

**Supplementary Figure 7.** Influence of multivitamin vs placebo on serum folate (N = 399).

**Supplementary Figure 8.** Hazard ratios and 95% confidence intervals for self-reported non-monitored outcomes according to randomized assignment, in intention-to-treat analyses.

**Supplementary Figure 9.** Hazard ratios and 95% confidence intervals for side effects according to randomized assignment, in intention-to-treat analyses.

**Supplementary Methods**

*Study Design*

Briefly, an embedded recruitment approach was conducted from June 2015 to March 2018 with mailings to 71,521 active participants in the Women’s Health Initiative (WHI) Extension Study,^1^ mailings by Brigham and Women’s Hospital (BWH) to 237,736 women and men contacted for but not randomized into the VITamin D and OmegA-3 TriaL,^2^ mass mailings to 2,616,343 U.S. women and men, and 3,380 volunteers who heard about the study through various sources. A total of 191,796 participants completed a brief initial screening questionnaire. To be eligible, participants agreed to forego multi-vitamin/multi-mineral (MVM) and cocoa supplement use during the trial, limit vitamin D to ≤1000 IU/day and calcium to ≤1200 mg/day from all supplemental sources and complete at least a 2-month placebo run-in phase taking ≥75% of the study pills. Safety exclusions included renal failure or dialysis, cirrhosis, and other serious conditions that would preclude participation. We also excluded participants with extreme sensitivity to caffeine, given the modest theobromine and caffeine content in the cocoa extract supplement.

A total of 35,669 eligible, willing, and consenting participants began a placebo run-in to eliminate poor compliers before randomization to increase study power.^3^ At the end of the run-in, participants returned a final compliance and eligibility questionnaire and a semi-quantitative food frequency questionnaire.^4^ In addition, 6,867 (32.0%) of randomized participants provided biospecimens prior to randomization and 603 in the greater Boston area completed a baseline clinic visit toward the end of the run-in phase with biospecimen collection and in-depth phenotyping.

From April 2016 to March 2018, 21,442 participants meeting all eligibility requirements were randomized to (1) active MVM and active cocoa extract, (2) active MVM and cocoa extract placebo, (3) active cocoa extract and MVM placebo, or (4) both placebos using a computer-generated permuted block approach blinded to trial investigators and stratified by sex (women, men), age (for women, 65-<70, 70-<75, 75-<80, 80-<85, and ≥85 years; for men, 60-<65, 65-<70, 70-<75, 75-<80, and ≥80 years), and recruitment source (WHI or BWH) in blocks of twelve. The algorithm used a randomized permuted block approach, stratified by sex, age group, and recruitment center (WHI or BWH). Participants within the same household were randomized to the same intervention whenever possible to reduce the risk of cross-contamination. The randomization schedule was prepared by a WHI Clinical Coordinating Center analyst and implemented by a WHI Clinical Coordinating Center software engineer. BWH staff then sent newly randomized COSMOS participants double-blinded calendar packs containing MVM or placebo tablets (and cocoa extract or placebo capsules).

*Primary and Secondary Outcomes*

For the MVM intervention, the primary outcome was total invasive cancer, excluding non-melanoma skin cancer. A key secondary outcome was a composite total cardiovascular disease (CVD) endpoint including incident myocardial infarction (MI), stroke, coronary revascularization, cardiovascular mortality, carotid artery surgery, peripheral artery surgery, and unstable angina requiring hospitalization. This was an expansion of our original primary CVD outcome definition of MI, stroke, coronary revascularization, and cardiovascular mortality because overall rates of CVD were adversely impacted by lower CVD rates possibly due to increasing use of statins and other preventive treatments, the influence of coronavirus disease 2019 (COVID-19) pandemic on 2020 CVD event rates, and a smaller proportion of older participants enrolled from WHI than anticipated.

Participants reporting a primary or secondary study outcome signed a medical record release form to request related medical records evaluated and processed based upon standardized WHI and BWH procedures.^5^ Outcomes were adjudicated by medical record review by a committee of physicians and investigators blinded to treatment assignment. Incident cancers were confirmed by a pathology report that substantiated a malignant primary invasive cancer at any location other than non-melanoma skin cancer;^5^ all histologic types and anatomic subsites were included. Noncancerous colorectal polyps, atypical benign breast disease, in situ cancers, and other premalignant benign conditions were excluded. Second primaries at a site and recurrences of cancer were also ascertained. MI, stroke,^6, 7^ coronary revascularization,^5^ and other vascular endpoints were confirmed using established clinical criteria. Unstable angina requiring hospitalization included reports of increased pain, use of medications to alleviate pain, no evidence of MI plus other related factors. Carotid artery surgery and peripheral artery surgery included review and identification from surgical and radiology reports. Coronary heart disease and stroke deaths were consistent with either outcome as an underlying cause. For participants determined to be deceased, we contacted next of kin to request permission to obtain medical records and a copy of the death certificate. For WHI participants, death certificates were alternatively requested from the state where the participant died. An outcomes committee reviewed records to assign cause of death. If records were unavailable (or participants were lost to follow-up), the National Death Index Plus was searched for cause of death according to the death-certificate information. Analyses included only confirmed outcomes.

*Statistical Analyses – Per-protocol and Biomarker Data*

Per-protocol analyses censored follow-up when the participant discontinued trial pills, began outside (non-study) use of a MVM, took outside (non-study) vitamin D > 1000 IU/day, and/or took approximately <75% of study pills (missed >8 days of study pills per month). Hazard ratios (HRs) and 95% confidence intervals (CIs) were then estimated using Cox regression models, weighted by the inverse probability of dependent-censoring for non-compliance and developed post hoc.^8^

For the biomarker analyses, first we explored the distribution of the biomarkers. The distribution of baseline 25-hydroxy-vitamin D (25(OH)D) (n = 399) was mostly symmetric and so data was analyzed on the untransformed scale. The distribution of baseline vitamin B_12_ was skewed so data was analyzed on the log-scale. There were 13 participants had vitamin B_12_ assay values of “>1500” assumed to be above 1500 pg/mL; these values were assigned a value of 1501 pg/mL. For serum folate, 48.9% of the cohort had values explicitly coded as “>22.3” ng/mL at baseline. Therefore, analyses examined the influence of MVM on serum folate coded folate as a binary variable (≤22.3 ng/mL vs. >22.3 ng/mL).

**Supplementary Results**

Of the participants who provided baseline and follow-up blood samples, we performed assays to measure changes in serum 25(OH)D, vitamin B_12_, and folate among 399 participants. Mean levels of 25(OH)D were balanced between MVM treatment arms at baseline (**Supplementary Table 1**). Serum 25(OH)D values were greater during follow-up for participants randomized to MVM versus placebo (overall difference in means 6.81 (5.55, 8.08) ng/mL after two years of follow-up) (**Supplementary Figure 5**). Due to the skewed distribution of vitamin B_12_ values, serum levels of vitamin B_12_ were log-transformed. At baseline, the median serum vitamin B_12_ values were balanced between arms (**Supplementary Table 1**). After two years of follow-up, the overall ratio of geometric means (95% CI) for the MVM group versus placebo was 1.44 (1.36, 1.54) (**Supplementary Figure 6**). For serum folate, 48.9% of the cohort had values >22.3 ng/ml at baseline. Therefore, folate was analyzed as a binary variable (≤22.3mg/mL versus >22.3 ng/mL). The proportion of individuals with folate values >22.3 mg/mL was balanced between arms (**Supplementary Table 1**). The overall risk ratio (95% CIs) for folate exceeding >22.3 ng/mL was 2.42 (2.09, 2.81) after two years of follow-up (**Supplementary Figure 7**).

**Supplementary Table 1. Characteristics of the participants at baseline, according to randomized assignment (n = 21,442)^1^**

**Supplementary Table 2. Vitamins and minerals contained in the COcoa Supplement and Multivitamin Outcomes Trial (COSMOS) versus Physicians’ Health Study II (PHS II) multi-vitamin/multi-mineral interventions.**

**Supplementary Figure 1. Cumulative hazard ratios of invasive cancer events^1^, according to year of follow-up, in multivitamin group and placebo group.**

^1^ Primary outcome: a composite of invasive cancers of any site other than non-melanoma skin cancer.

Summary statistics were from Cox regression models that stratified baseline hazard functions by cocoa extract trial randomization group, age, sex, and recruitment cohort (intention-to-treat analyses). P-value was for the effect of randomization group, based on a stratified score (log-rank) test.

Kaplan-Meier estimates were complemented by cumulative HRs (95% CI; lower panel), computed under the proportional hazards assumption, using increasingly longer cumulative follow-up elapsed from randomization. For example, if the trial had ended after only 2 years of follow-up, the resulting HR (95% CI) = 1.00 (0.85, 1.17) for 296 vs 297 events. The blue reference line indicates the estimated HR = 0.97 for total cumulative follow-up.

**Supplementary Figure 2. Hazard ratios^1^ of the primary outcome^2^ according to subgroup, comparing multivitamin group with placebo group.**

^1^ Summary statistics were from Cox regression models that stratified baseline hazard functions by cocoa extract trial randomization group, age, sex, recruitment cohort and subgroup. P-value was for the interaction (product term) between randomization group and participant characteristic based on a stratified score (log-rank) test; 1–degree-of-freedom score tests for trend were used for age group and fruit and vegetable intake. Analyses were not adjusted for multiple comparisons.

Participants were missing baseline data for ever smoked (n = 311, 1.5%), statin use (n=268, 1.2%), aspirin use (n=206, 1.0%), fruit and vegetable intake (n = 1539, 7.2%) and prior use of any dietary supplement (n = 115, 0.5%).

^2^ This outcome was a composite of invasive cancers of any site other than non-melanoma skin cancer.

^3^ History of cancer (excluding non-melanoma skin cancer) at baseline.

^4^ Prior use of multivitamins, vitamin D or calcium supplements at baseline.

^5^ Cocoa extract.

**Supplementary Figure 3. Proportion of participants who reported abstaining from use of non-study multivitamins, abstaining from use of non-study vitamin D > 1000 IU/day, compliance to study pills, total compliance (adherence to all previous components),^1^ and total compliance and completion of follow-up questionnaires**^2^ **during the intervention phase (N=21,442).**

Dot (whisker) plots summarize proportion of participants that reported compliance (95% CI) for each semi-annual questionnaire during follow-up. Only compliance to study pills was assessed at 18 months and close-out (red).

^1^ Total compliance was defined as self-report of having missed ≤ 8 days of multivitamin study pills per month and did not take personal non-study multivitamin pills or non-study vitamin D > 1000 IU/day. Participants with total compliance were compliant for all components, so could not be computed at 18 months or at closeout.

^2^ Participants not known to be deceased and willing to complete questionnaires within 2 months of the corresponding time point.

^3^ Use of personal non-study multivitamin was not assessed at 18 months or at closeout, so total compliance^1^ could not be computed either.

^4^ Study closeout occurred on December 31, 2020, so length of time from randomization to close-out varied; median (Q1 – Q3) = 43.5 (39.7 – 50.3) months.

**Supplementary Figure 4. Hazard ratios and 95% confidence intervals^1^ for the primary and secondary cancer outcomes, according to randomized assignment, where follow-up of noncompliant participants was censored^2^.**

^1^ Summary statistics were from weighted Cox regression models that stratified baseline hazard functions by cocoa extract trial randomization group, age, sex, and recruitment cohort, and used the robust sandwich estimator for variance. Time-dependent weights were the inverse probability of compliance^2^, where probabilities were estimated from a Cox regression model with baseline hazard functions stratified by age, sex, recruitment source, multivitamin trial arm and cocoa extract trial arm, and included baseline history of cancer, family history of cancer, prior use of MVM, calcium or vitamin D supplements, smoking status and the interaction between smoking and randomization group as covariates. P-values were not adjusted for multiple comparisons.

^2^ A participant’s follow-up was censored at the first time they reported having missed >8 days of multivitamin study pills per month, took personal non-study multivitamins, took personal non-study vitamin D > 1000 IU/day, or did not respond to a semiannual questionnaire.

^3^ This outcome was a composite of invasive cancers of any site other than non-melanoma skin cancer.

**Supplementary Figure 5. Influence of multivitamin vs placebo on serum 25−hydroxyvitamin D (N = 399).** Mean (95% CI; bold red and blue dot and whiskers) and overall treatment effect were estimated via linear mixed effects models that included a common intercept for randomization groups, indicator variables for visit (1 or 2), interactions between randomization group x visit, covariate adjustment for sex and baseline age, and an unstructured variance-covariance matrix. The overall difference assumed a constant treatment effect.

**Supplementary Figure 6. Influence of multivitamin vs placebo on serum vitamin B_12_ (N = 399).** Geometric mean (95% CI; bold red and blue dots and whiskers) and overall treatment effect were estimated via linear mixed effects models that included a common intercept for randomization groups, indicator variables for visit (1 or 2), interactions between randomization group x visit, covariate adjustment for sex and baseline age, and an unstructured variance-covariance; vitamin B_12_ was log-transformed. The overall difference assumed a constant treatment effect.

**Supplementary Figure 7. Influence of multivitamin vs placebo on serum folate (N = 399).** Probability of folate measurements exceeding 22.3 ng/mL (95% CI; bold red and blue dots and whiskers) and overall treatment effect were estimated via longitudinal Poisson regression models that included a common intercept for randomization groups, indicator variables for visit (1 or 2), interactions between randomization group x visit, covariate adjustment for sex and baseline age, and within-participants correlations were unstructured. Parameters were estimated via generalized estimating equations using a robust sandwich estimator for variance. Folate was coded as binary response variable (0 = folate ≤ 22.3 ng/mL; 1 = folate > 22.3 ng/mL). The overall difference assumed a constant treatment effect.

**Supplementary Figure 8. Hazard ratios and 95% confidence intervals^1^ for self-reported non-monitored outcomes^2^ according to randomized assignment, in intention-to-treat analyses.**

^1^ Summary statistics were from Cox regression models that stratified baseline hazard functions by cocoa extract trial randomization group, age, sex, and recruitment cohort. P-values were not adjusted for multiple comparisons.

^2^ Collected from all COSMOS trial participants at 12, 24, 36, 48 months and study closeout; self-reports from WHI participants were augmented with WHI data. Participants were asked to report only new diagnoses within the last year. Does not exclude participants that may have reported a prevalent condition at baseline.

^3^ Not collected at 12 months. Summary statistics adjusted for the shorter follow-up.

**Supplementary Figure 9. Hazard ratios and 95% confidence intervals^1^ for side effects^2^ according to randomized assignment, in intention-to-treat analyses.**

^1^ Summary statistics were from Cox regression models that stratified baseline hazard functions by cocoa extract trial randomization group, age, sex, and recruitment cohort. P-values were not adjusted for multiple comparisons.

^2^ Reports at 6, 18, 30 and 42 months reflect symptoms experienced in the last 6 months, while reports at 12, 24, 36, 48 months and study closeout reflect symptoms experienced over the past year.

**Supplementary Table 1. Characteristics of the participants at baseline, according to randomized assignment (N = 21,442)^1^**

|  | **Total**  **(N = 21,442)** | | **Multivitamin**  **(N = 10,720)** | | **Placebo**  **(N = 10,722)** | |
| --- | --- | --- | --- | --- | --- | --- |
|  | **N** | **%** | **N** | **%** | **N** | **%** |
| Female sex – no. (%) | 12666 | (59.1) | 6338 | (59.1) | 6328 | (59.0) |
| Age – y | 72.1 | ±6.6 | 72.1 | ±6.6 | 72.1 | ±6.6 |
| Age group, y – no. (%) |  |  |  |  |  |  |
| 60 – 64 | 2705 | (12.6) | 1351 | (12.6) | 1354 | (12.6) |
| 65 – 69 | 6519 | (30.4) | 3260 | (30.4) | 3259 | (30.4) |
| 70 – 74 | 5774 | (26.9) | 2886 | (26.9) | 2888 | (26.9) |
| 75 – 79 | 3751 | (17.5) | 1875 | (17.5) | 1876 | (17.5) |
| 80 – 84 | 1761 | (8.2) | 889 | (8.3) | 872 | (8.1) |
| 85 – 89 | 741 | (3.5) | 366 | (3.4) | 375 | (3.5) |
| ≥ 90 | 191 | (0.9) | 93 | (0.9) | 98 | (0.9) |
| Recruitment source – no. (%) |  |  |  |  |  |  |
| WHI | 4611 | (21.5) | 2307 | (21.5) | 2304 | (21.5) |
| VITAL | 6943 | (32.4) | 3492 | (32.6) | 3451 | (32.2) |
| Other | 9888 | (46.1) | 4921 | (45.9) | 4967 | (46.3) |
| Hispanic/Latino – no. (%) ^2^ | 544 | (2.6) | 284 | (2.8) | 260 | (2.5) |
| Race – no. (%) ^2^ |  |  |  |  |  |  |
| White | 19294 | (90.0) | 9628 | (89.8) | 9666 | (90.2) |
| African American | 1131 | (5.3) | 568 | (5.3) | 563 | (5.3) |
| Asian/Pacific Islander | 499 | (2.3) | 258 | (2.4) | 241 | (2.2) |
| American Indian/Alaska Native | 59 | (0.3) | 37 | (0.3) | 22 | (0.2) |
| Multiracial/other/unknown or not reported | 459 | (2.1) | 229 | (2.1) | 230 | (2.1) |
| Hispanic/Latino – no. (%) ^2^ | 544 | (2.6) | 284 | (2.8) | 260 | (2.5) |
| HS diploma/GED or less | 2296 | (10.8) | 1180 | (11.1) | 1116 | (10.5) |
| Attended or graduated from college | 8685 | (40.9) | 4315 | (40.7) | 4370 | (41.1) |
| Post-college | 10241 | (48.3) | 5104 | (48.2) | 5137 | (48.4) |
| Smoking status – no. (%) |  |  |  |  |  |  |
| Never | 11565 | (54.7) | 5808 | (54.9) | 5757 | (54.5) |
| Past | 8731 | (41.3) | 4345 | (41.1) | 4386 | (41.5) |
| Current | 835 | (4.0) | 417 | (3.9) | 418 | (4.0) |
| Total MET-hours per week from exercise – median [interquartile range] | 17.1 | [5.2- 32.8] | 17.5 | [5.3- 33.0] | 16.5 | [5.1- 32.6] |
| Body mass index, kg/m^2^ – median [interquartile range] ^3^ | 26.8 | [24.0- 30.4] | 26.7 | [24.0- 30.3] | 26.9 | [24.0- 30.5] |
| Body mass index, kg/m^2^ – no. (%) ^3^ |  |  |  |  |  |  |
| < 25 | 7070 | (33.6) | 3533 | (33.7) | 3537 | (33.6) |
| 25-<30 | 8230 | (39.2) | 4166 | (39.7) | 4064 | (38.6) |
| ≥ 30 | 5718 | (27.2) | 2800 | (26.7) | 2918 | (27.7) |
| Fruit and vegetable intake – no. (%) |  |  |  |  |  |  |
| < 4 serving/day | 6973 | (35.0) | 3534 | (35.4) | 3439 | (34.7) |
| 4-<7 servings/day | 7897 | (39.7) | 3935 | (39.4) | 3962 | (40.0) |
| ≥ 7 servings/day | 5033 | (25.3) | 2518 | (25.2) | 2515 | (25.4) |
| Multivitamin use before run-in – no. (%) | 8795 | (41.2) | 4413 | (41.3) | 4382 | (41.0) |
| Vitamin D from supplements before run-in – no. (%) |  |  |  |  |  |  |
| None | 7960 | (37.6) | 4012 | (37.9) | 3948 | (37.3) |
| ≤ 1000 IU/day | 8670 | (41.0) | 4331 | (40.9) | 4339 | (41.0) |
| > 1000 IU/day | 4536 | (21.4) | 2238 | (21.2) | 2298 | (21.7) |
| Calcium from supplements before run-in – no. (%) |  |  |  |  |  |  |
| None | 10917 | (51.5) | 5483 | (51.8) | 5434 | (51.3) |
| ≤ 1200 mg/day | 9200 | (43.4) | 4596 | (43.4) | 4604 | (43.5) |
| > 1200 mg/day | 1066 | (5.0) | 516 | (4.9) | 550 | (5.2) |
| Cocoa extract use before run-in – no. (%) | 91 | (0.4) | 42 | (0.4) | 49 | (0.5) |
| Statin use – no. (%) | 8911 | (42.1) | 4464 | (42.2) | 4447 | (41.9) |
| Aspirin use – no. (%) | 10379 | (48.9) | 5168 | (48.7) | 5211 | (49.1) |
| NSAID use – no. (%) | 6143 | (29.0) | 3020 | (28.6) | 3123 | (29.5) |
| History of diabetes – no. (%) | 2864 | (13.4) | 1415 | (13.2) | 1449 | (13.5) |
| History of high blood pressure – no. (%) | 12423 | (58.1) | 6153 | (57.6) | 6270 | (58.6) |
| Current use of medication for high blood pressure – no. (%) | 11217 | (53.0) | 5572 | (52.7) | 5645 | (53.2) |
| Systolic blood pressure, mmHg – no. (%) ^3^ |  |  |  |  |  |  |
| < 120 | 6049 | (32.4) | 3007 | (32.3) | 3042 | (32.5) |
| 120-139 | 10793 | (57.8) | 5393 | (58.0) | 5400 | (57.7) |
| ≥ 140 | 1817 | (9.7) | 899 | (9.7) | 918 | (9.8) |
| Diastolic blood pressure, mmHg – no. (%) ^3^ |  |  |  |  |  |  |
| < 80 | 13708 | (73.8) | 6854 | (74.0) | 6854 | (73.6) |
| 80-89 | 4401 | (23.7) | 2163 | (23.4) | 2238 | (24.0) |
| ≥ 90 | 465 | (2.5) | 244 | (2.6) | 221 | (2.4) |
| History of revascularization (CABG/PCI) – no. (%) | 862 | (4.0) | 426 | (4.0) | 436 | (4.1) |
| History of unstable angina – no. (%) | 374 | (1.8) | 194 | (1.8) | 180 | (1.7) |
| History of carotid artery surgery/stenting – no. (%) | 93 | (0.4) | 44 | (0.4) | 49 | (0.5) |
| History of peripheral artery surgery/stenting – no. (%) | 144 | (0.7) | 81 | (0.8) | 63 | (0.6) |
| History of heart failure – no. (%) | 364 | (1.7) | 188 | (1.8) | 176 | (1.7) |
| History of cancer excluding non-melanoma skin cancer – no. (%) | 3550 | (16.6) | 1813 | (16.9) | 1737 | (16.2) |
| Screening behaviors in the past 10 years |  |  |  |  |  |  |
| Colonoscopy or sigmoidoscopy | 1.0 | [1.0 – 2.0] | 1.0 | [1.0 – 2.0] | 1.0 | [1.0 – 2.0] |
| Mammogram (females only) | 5.0 | [4.0 – 5.0] | 5.0 | [4.0 – 5.0] | 5.0 | [4.0 – 5.0] |
| Prostate-specific antigen test (males only) | 4.0 | [1.0 – 5.0] | 4.0 | [1.0 – 5.0] | 4.0 | [1.0 – 5.0] |
| Family history of cancer – no.(%) ^4^ | 9220 | (45.1) | 4603 | (45.0) | 4617 | (45.2) |
| History of macular degeneration – no. (%) | 623 | (3.0) | 322 | (3.1) | 301 | (2.9) |
| History of cataract – no. (%) | 8629 | (40.8) | 4317 | (40.8) | 4312 | (40.8) |
| History of cataract surgery – no. (%) | 6413 | (30.2) | 3199 | (30.2) | 3214 | (30.3) |
| Baseline serum biomarkers ^5^ |  |  |  |  |  |  |
| 25-hydroxy-vitamin D (25(OH)D) – mean (±SD), ng/mL | 31.3 | (9.0) | 31.6 | (9.1) | 31.0 | (8.9) |
| Vitamin B_12_ – median (Q1 – Q3), pg/mL | 316.0 | [235.0 - 443.0] | 321.5 | [234.0 - 436.0] | 315.0 | [235.0 - 455.0] |
| Folate > 22.3 ng/mL – no. (%) | 195 | (48.9) | 99 | (49.0) | 96 | (48.7) |

^1^ Plus–minus values are means ±SD. Medians [interquartile range] summarize skewed variables. Percentages may not sum to 100 because of rounding. Data on age and sex were complete.

^2^ Ethnic group and race were self-reported by participants. Multiracial participants self-identified with more than one race. Participants of other race or unknown race self-identified with those categories.

^3^ Self-reported.

^4^ Family history of cancer included lung, colorectal, breast (female participants) and prostate cancer (male participants).

^5^ Serum biomarkers were measured on the multivitamin biospecimen-subcohort. There were N = 399 (202 in active MVM vs 197 in placebo MVM) randomly selected participants with baseline and at least one follow-up measurement.

**Supplementary Table 2. Vitamins and minerals contained in the COcoa Supplement and Multivitamin Outcomes Trial (COSMOS) versus Physicians’ Health Study II (PHS II) multi-vitamin/multi-mineral interventions.**

| **Vitamin or mineral** | **COSMOS**  **Centrum Silver** | **PHS II**  **Centrum Silver** |
| --- | --- | --- |
| Vitamin A (IU) | 2500  40% β-carotene | 5000  50% β-carotene |
| Vitamin C (mg) | 60 | 60 |
| Vitamin D (IU) | 1000 | 400 |
| Vitamin E (IU) | 50 | 45 |
| Vitamin K (µg) | 30 | 10 |
| Thiamin (mg) | 1.5 | 1.5 |
| Riboflavin (mg) | 1.7 | 1.7 |
| Niacin (mg) | 20 | 20 |
| Vitamin B_6_ (mg) | 3 | 3 |
| Folic Acid (µg) | 400 | 400 |
| Vitamin B_12_ (µg) | 25 | 25 |
| Biotin (µg) | 30 | 30 |
| Pantothenic Acid (mg) | 10 | 10 |
| Calcium (mg) | 220 | 200 |
| Iron (mg) | - | 4 |
| Phosphorus (mg) | 20 | 48 |
| Iodine (µg) | 150 | 150 |
| Magnesium (mg) | 50 | 100 |
| Zinc (mg) | 11 | 15 |
| Selenium (µg) | 19 | 20 |
| Copper (mg) | 0.5 | 2 |
| Manganese (mg) | 2.3 | 3.5 |
| Chromium (µg) | 50 | 130 |
| Molybdenum (µg) | 45 | 160 |
| Chloride (mg) | 72 | 72.6 |
| Potassium (mg) | 80 | 80 |
| Boron (µg) | - | 150 |
| Nickel (µg) | 5 | 5 |
| Vanadium (µg) | 10 | 10 |
| Silicon (mg) | 2 | 2 |
| Lutein (µg) | 250 | - |
| Lycopene (µg) | 300 | - |

**Supplementary Figure 1. Cumulative hazard ratios of invasive cancer events^1^, according to year of follow-up, in multivitamin group and placebo group (N =21,442).**


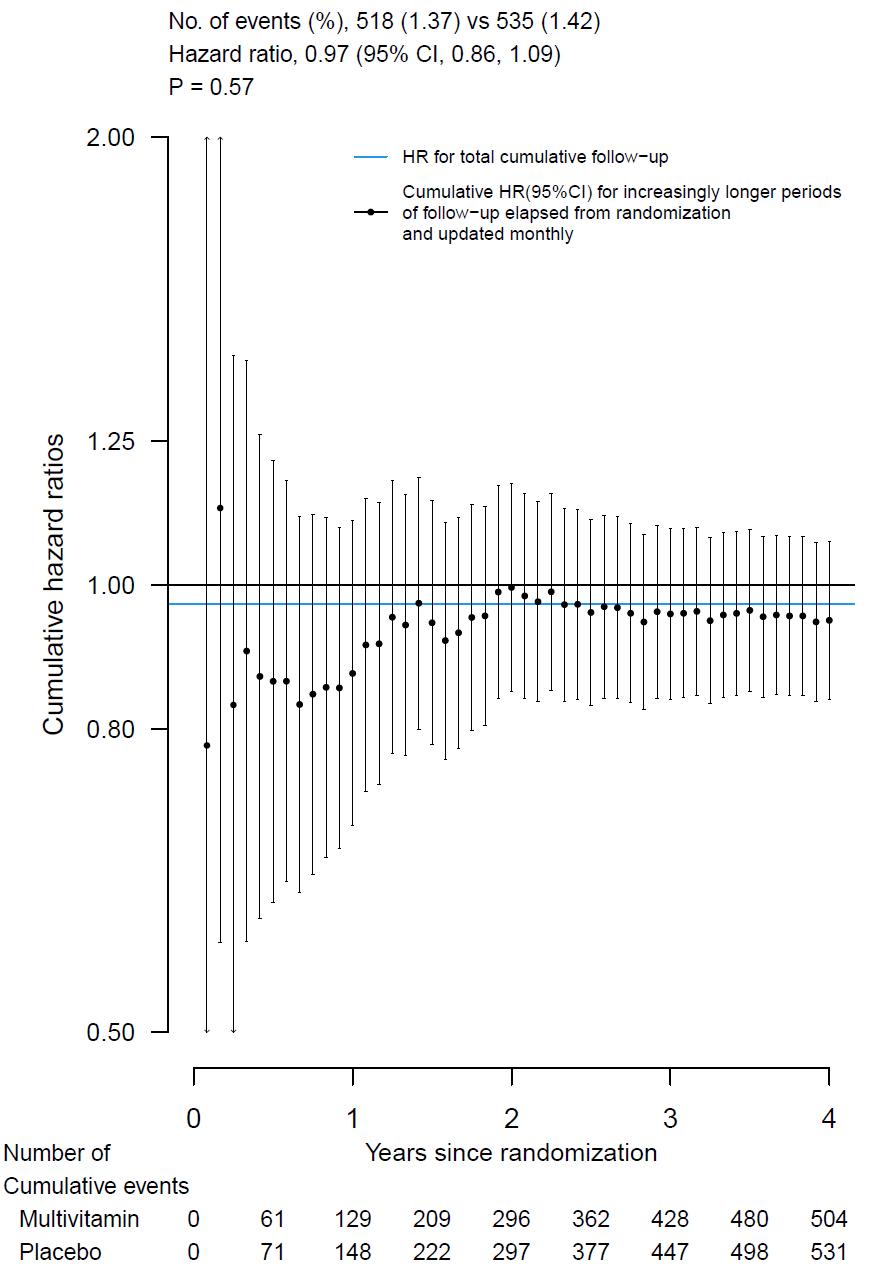


**Supplementary Figure 2. Hazard ratios and 95% confidence interval^1^ for the primary outcome^2^ according to subgroup, comparing multivitamin group with placebo group.**


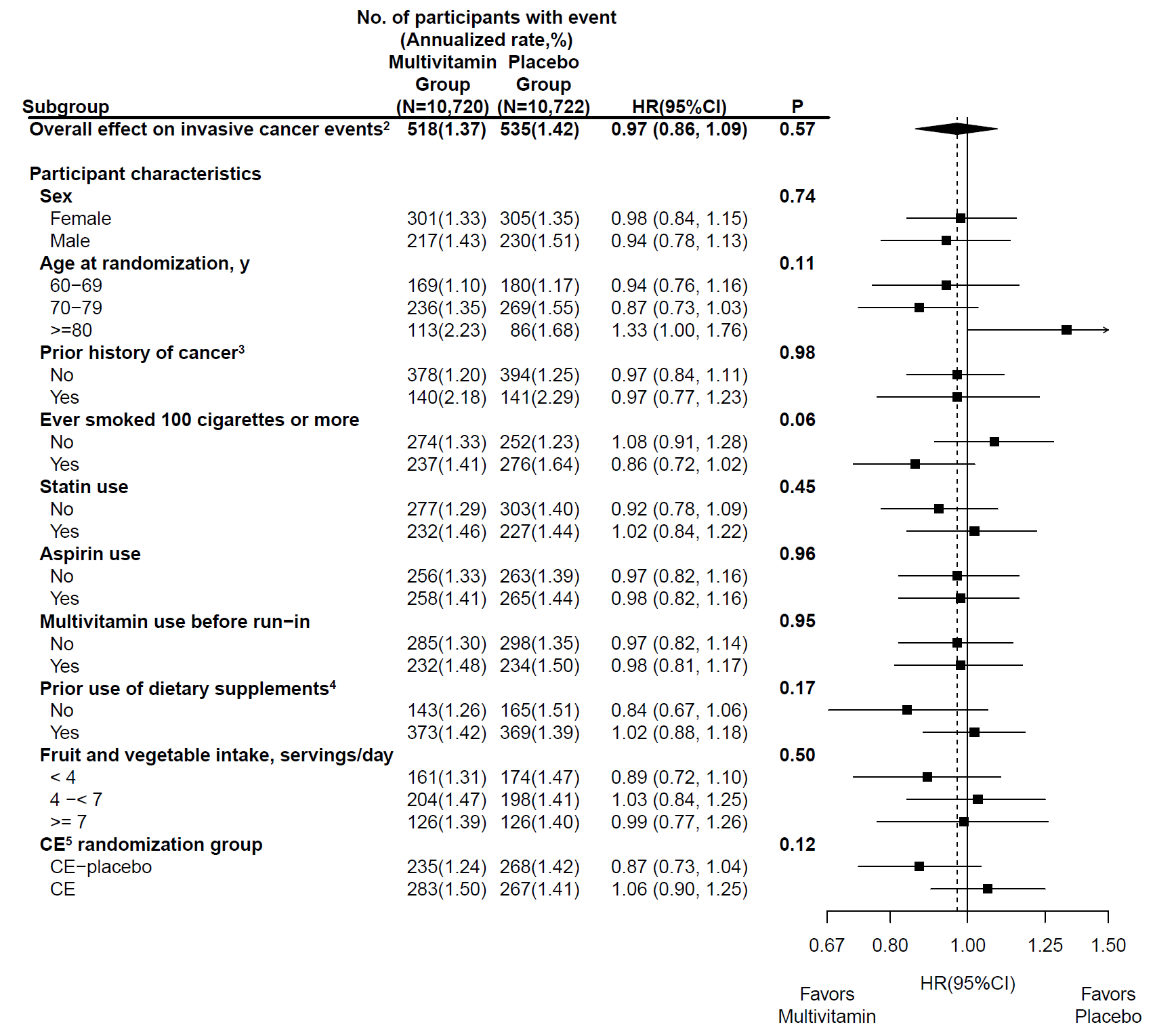


**Supplementary Figure 3. Proportion of participants who reported abstaining from use of non-study multivitamins, abstaining from use of non-study vitamin D > 1000 IU/day, compliance to study pills, total compliance (adherence to all previous components),^1^ and total compliance and completion of follow-up questionnaires^2^ during the intervention phase (N=21,442).**


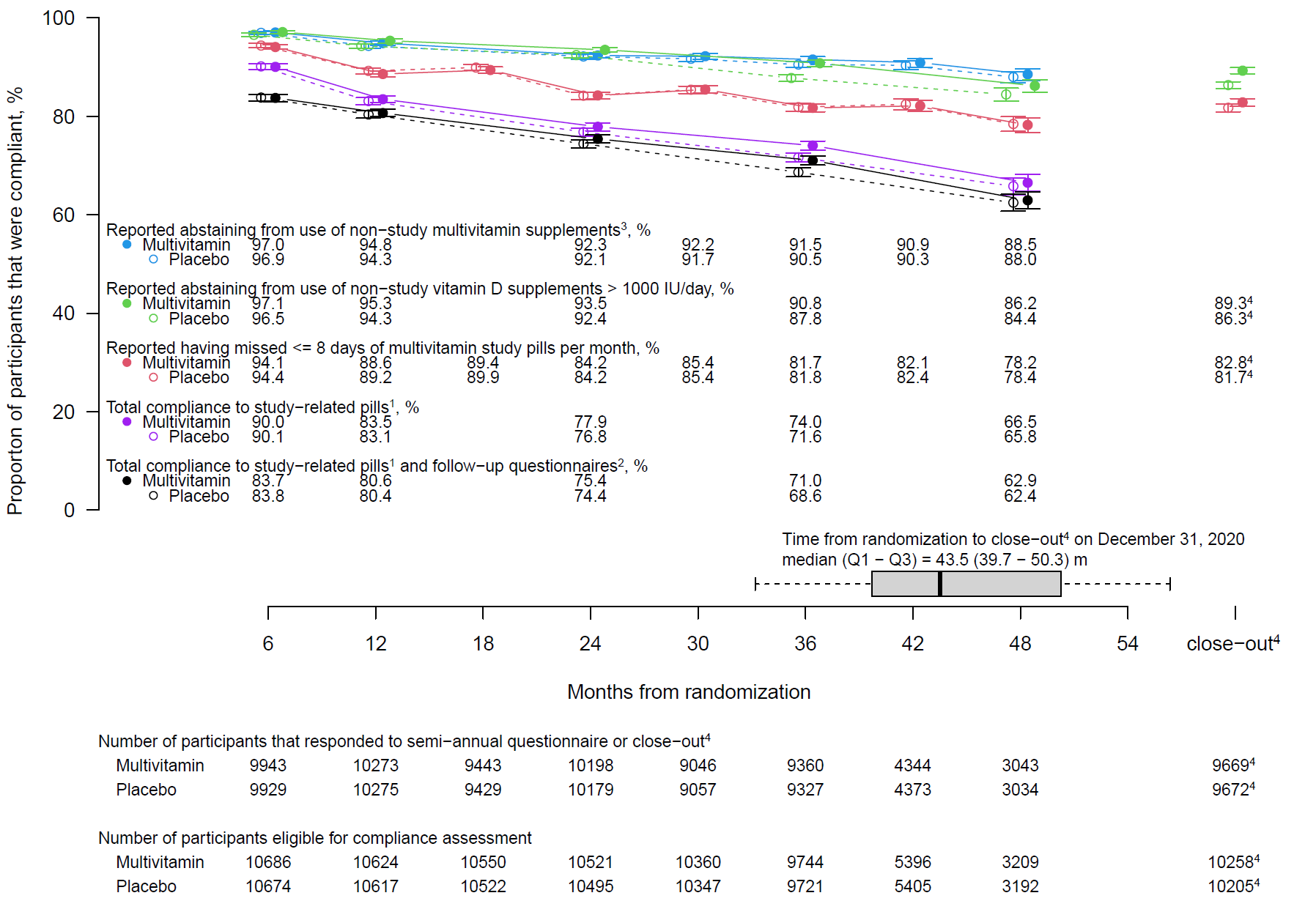


**Supplementary Figure 4. Hazard ratios and 95% confidence intervals^1^ for the primary and secondary cancer outcomes, according to randomized assignment, where follow-up of noncompliant participants was censored^2^.**


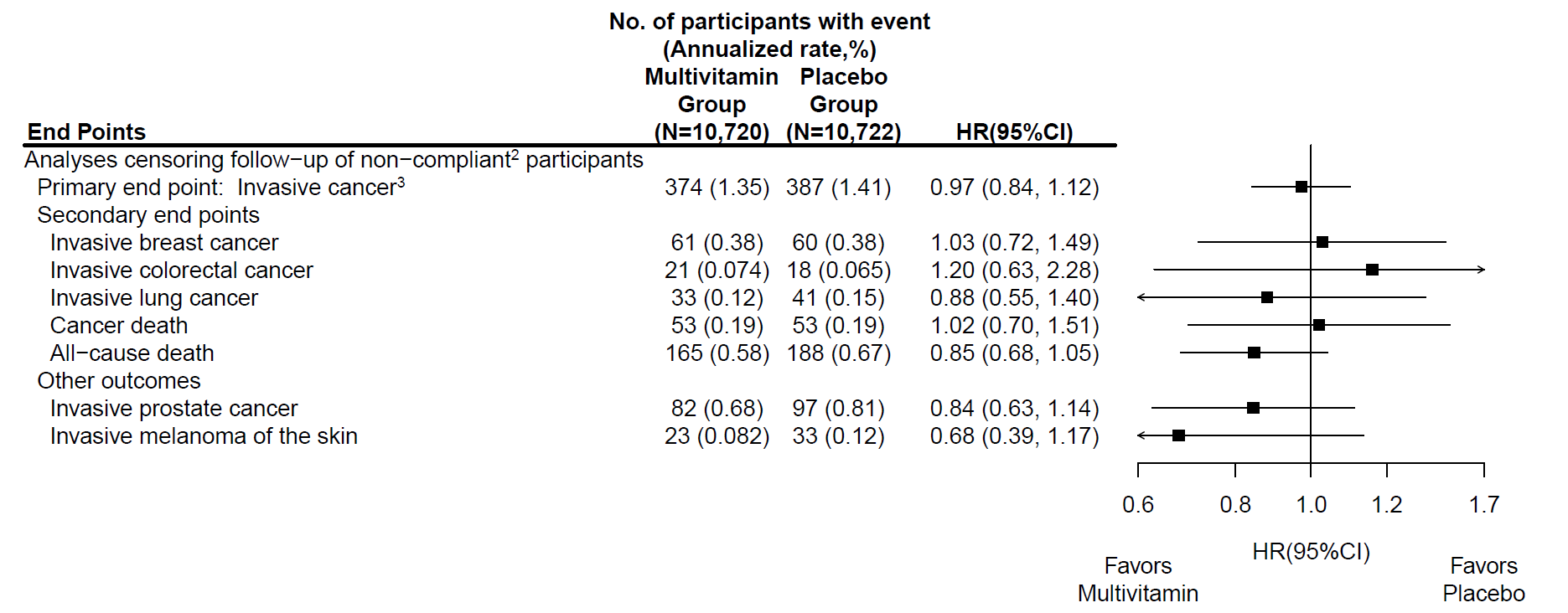


**Supplementary Figure 5. Influence of multivitamin vs placebo on serum 25−hydroxyvitamin D (N = 399).**


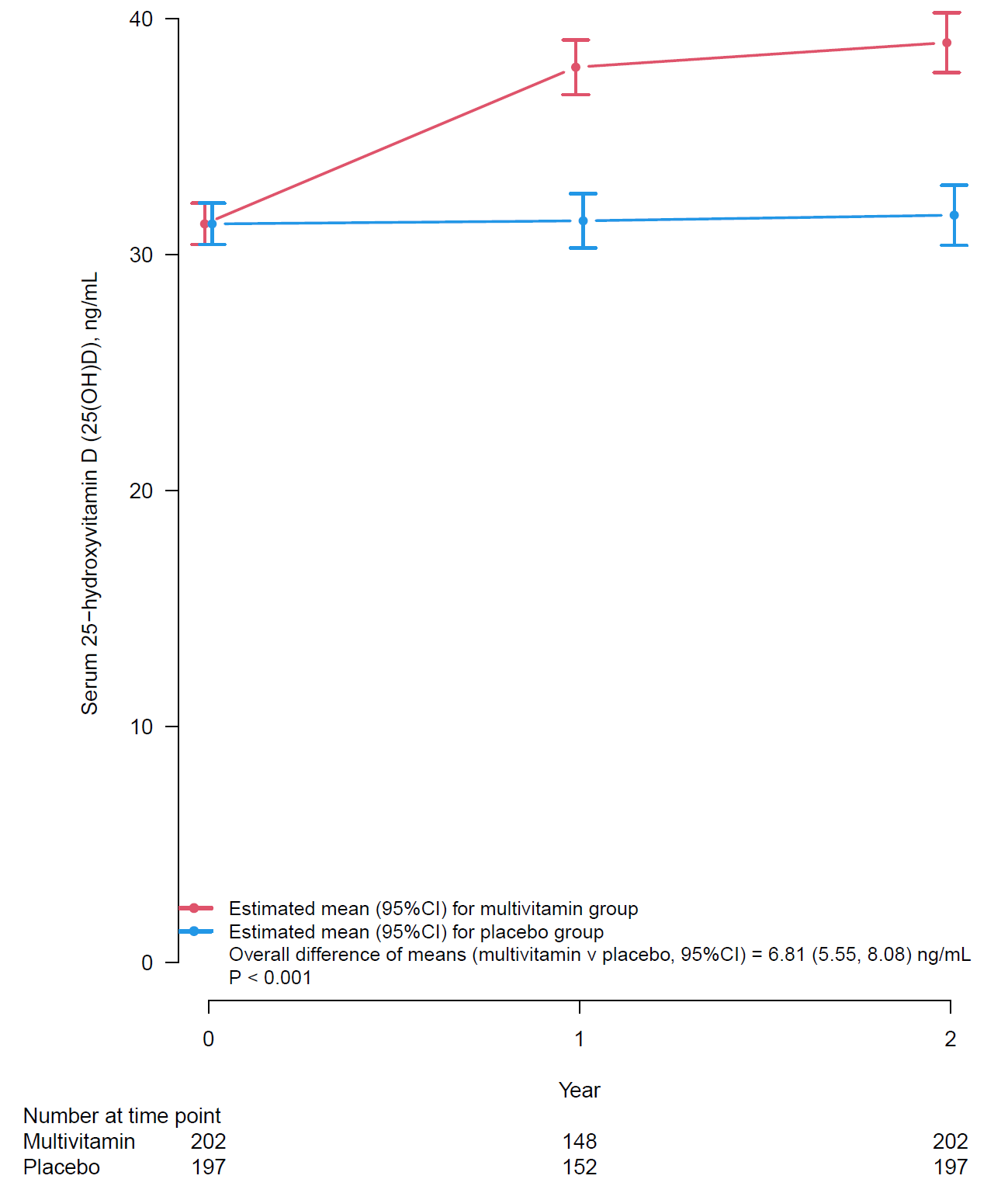


**Supplementary Figure 6. Influence of multivitamin vs placebo on serum vitamin B_12_ (N = 399).**


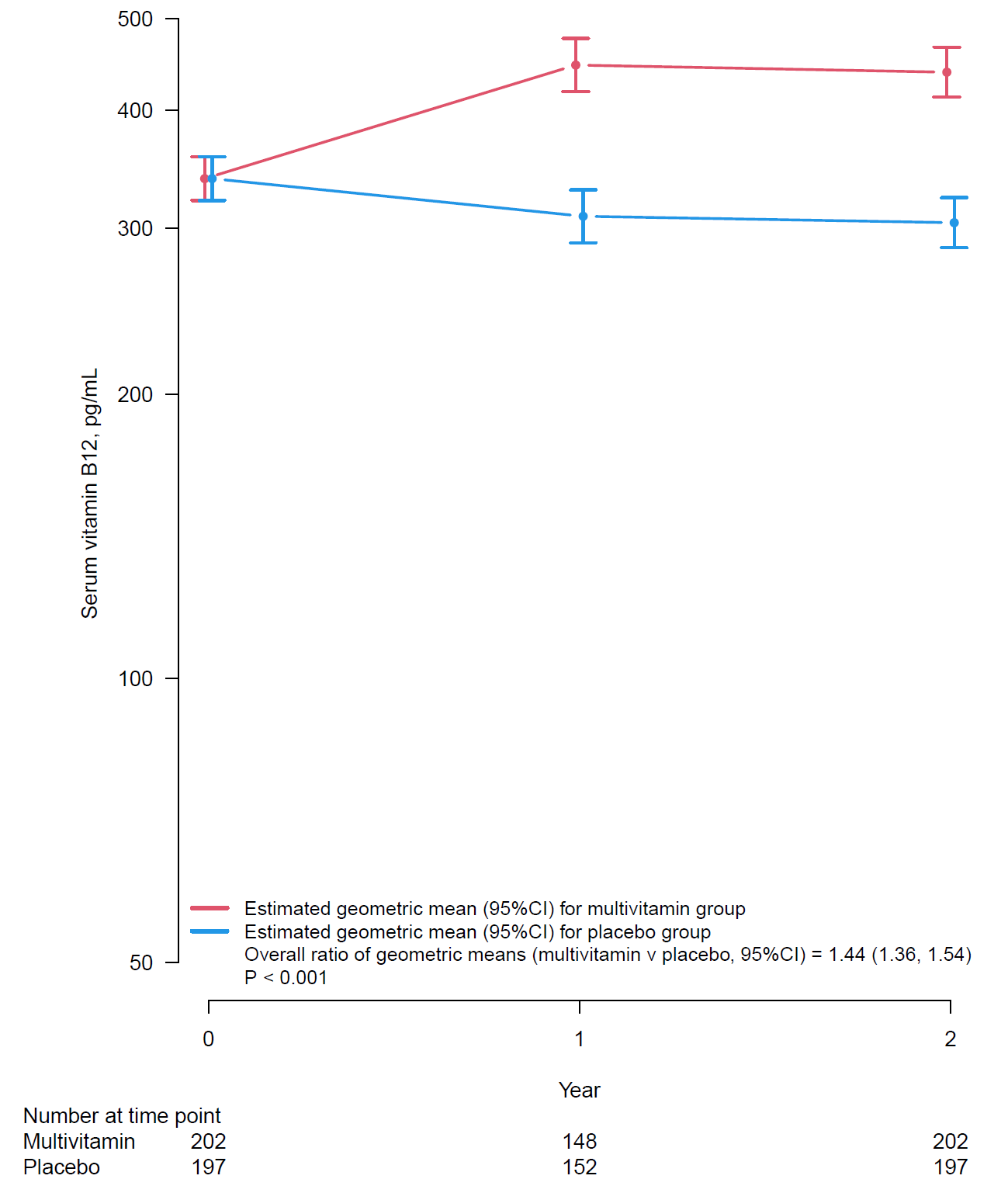


**Supplementary Figure 7. Influence of multivitamin vs placebo on serum folate (N = 399).**


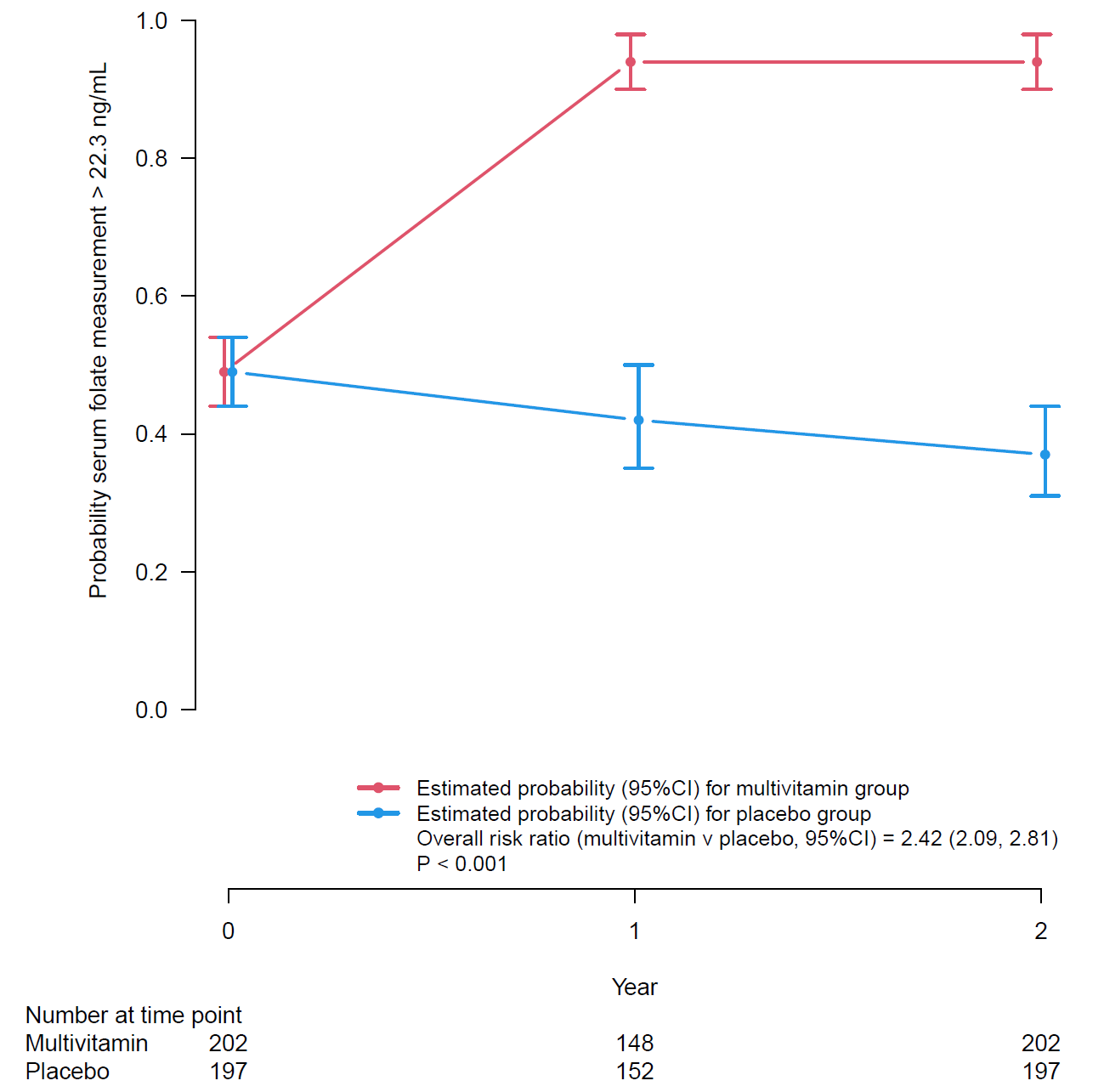


**Supplementary Figure 8. Hazard ratios and 95% confidence intervals^1^ for self-reported non-monitored outcomes^2^ according to randomized assignment, in intention-to-treat analyses.**


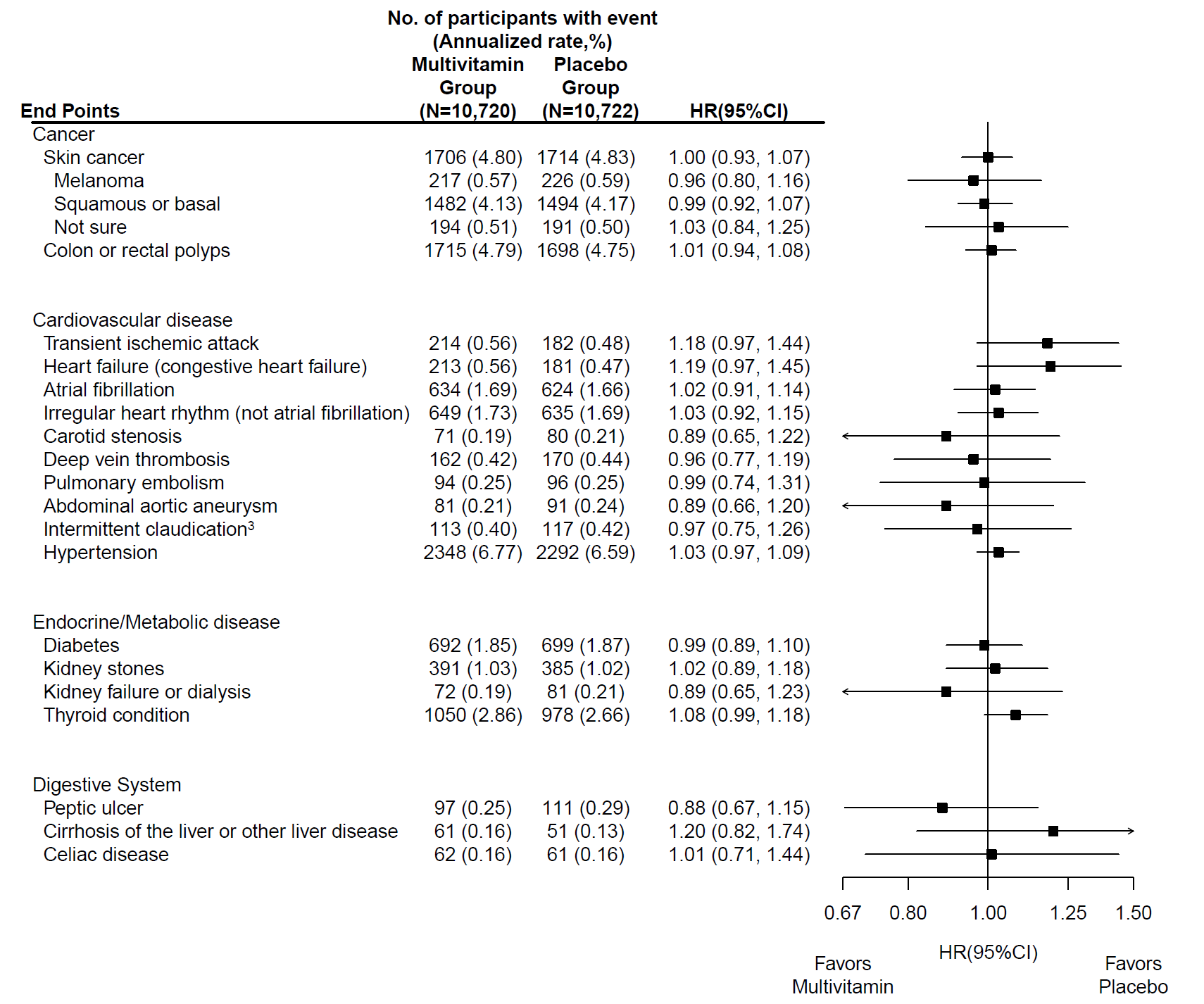


**Supplementary Figure 9. Hazard ratios and 95% confidence intervals^1^ for side effects^2^ according to randomized assignment, in intention-to-treat analyses.**


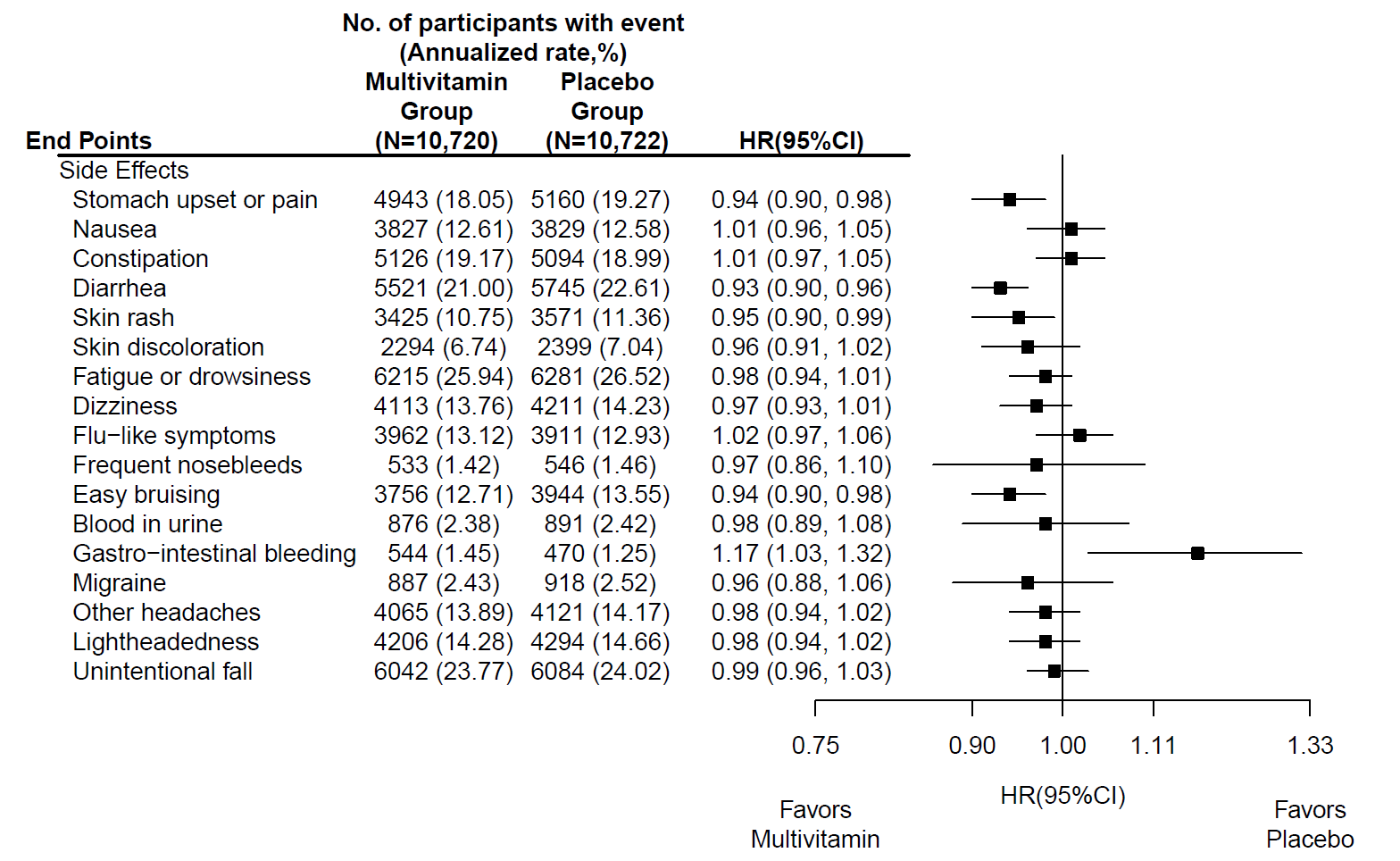


**References**

1. Cauley JA, Crandall C. The Women's Health Initiative: a landmark resource for skeletal research since 1992. *J Bone Mineral Res*. 2020;35(5):845-860. doi:10.1002/jbmr.4026

2. Bassuk SS, Manson JE, Lee IM, et al. Baseline characteristics of participants in the VITamin D and OmegA-3 TriaL (VITAL). *Contemporary clinical trials*. Mar 2016;47:235-43. doi:10.1016/j.cct.2015.12.022

3. Lang JM, Buring JE, Rosner B, Cook N, Hennekens CH. Estimating the effect of the run-in on the power of the Physicians' Health Study. *Stat Med*. Oct 1991;10(10):1585-93.

4. Feskanich D, Rimm EB, Giovannucci EL, et al. Reproducibility and validity of food intake measurements from a semiquantitative food frequency questionnaire. *J Am Diet Assoc*. Jul 1993;93(7):790-6.

5. Curb JD, McTiernan A, Heckbert SR, et al. Outcomes ascertainment and adjudication methods in the Women's Health Initiative. *Ann Epidemiol*. Oct 2003;13(9 Suppl):S122-8. doi:S1047279703000486 [pii]

6. Adams HP, Jr., Bendixen BH, Kappelle LJ, et al. Classification of subtype of acute ischemic stroke. Definitions for use in a multicenter clinical trial. TOAST. Trial of Org 10172 in Acute Stroke Treatment. *Stroke*. Jan 1993;24(1):35-41.

7. Bamford J, Sandercock P, Dennis M, Burn J, Warlow C. Classification and natural history of clinically identifiable subtypes of cerebral infarction. *Lancet*. Jun 22 1991;337(8756):1521-6.

8. Robins JM, Finkelstein DM. Correcting for noncompliance and dependent censoring in an AIDS Clinical Trial with inverse probability of censoring weighted (IPCW) log-rank tests. *Biometrics*. Sep 2000;56(3):779-88. doi:10.1111/j.0006-341x.2000.00779.x
